# Human cell-based *in vitro* systems to assess respiratory toxicity: a case study using silanes

**DOI:** 10.1093/toxsci/kfad074

**Published:** 2023-07-27

**Authors:** Monita Sharma, Andreas O Stucki, Sandra Verstraelen, Todd J Stedeford, An Jacobs, Frederick Maes, David Poelmans, Jo Van Laer, Sylvie Remy, Evelien Frijns, David G Allen, Amy J Clippinger

**Affiliations:** PETA Science Consortium International e.V., 70499 Stuttgart, Germany; PETA Science Consortium International e.V., 70499 Stuttgart, Germany; Sustainable HEALTH Unit, Flemish Institute for Technological Research (VITO), BE-2400 Mol, Belgium; The Acta Group, Washington, District of Columbia 20037, USA; Sustainable HEALTH Unit, Flemish Institute for Technological Research (VITO), BE-2400 Mol, Belgium; Sustainable HEALTH Unit, Flemish Institute for Technological Research (VITO), BE-2400 Mol, Belgium; Sustainable HEALTH Unit, Flemish Institute for Technological Research (VITO), BE-2400 Mol, Belgium; Sustainable HEALTH Unit, Flemish Institute for Technological Research (VITO), BE-2400 Mol, Belgium; Sustainable HEALTH Unit, Flemish Institute for Technological Research (VITO), BE-2400 Mol, Belgium; Sustainable HEALTH Unit, Flemish Institute for Technological Research (VITO), BE-2400 Mol, Belgium; Inotiv, Research Triangle Park, North Carolina 27560, USA; PETA Science Consortium International e.V., 70499 Stuttgart, Germany

**Keywords:** new approach methodologies (NAMs), nonanimal methods, inhalation, respiratory toxicity, in vitro methods

## Abstract

Inhalation is a major route by which human exposure to substances can occur. Resources have therefore been dedicated to optimize human-relevant *in vitro* approaches that can accurately and efficiently predict the toxicity of inhaled chemicals for robust risk assessment and management. In this study—the *IN vitro* Systems to PredIct REspiratory toxicity Initiative—2 cell-based systems were used to predict the ability of chemicals to cause portal-of-entry effects on the human respiratory tract. A human bronchial epithelial cell line (BEAS-2B) and a reconstructed human tissue model (MucilAir, Epithelix) were exposed to triethoxysilane (TES) and trimethoxysilane (TMS) as vapor (mixed with N_2_ gas) at the air-liquid interface. Cell viability, cytotoxicity, and secretion of inflammatory markers were assessed in both cell systems and, for MucilAir tissues, morphology, barrier integrity, cilia beating frequency, and recovery after 7 days were also examined. The results show that both cell systems provide valuable information; the BEAS-2B cells were more sensitive in terms of cell viability and inflammatory markers, whereas MucilAir tissues allowed for the assessment of additional cellular effects and time points. As a proof of concept, the data were also used to calculate human equivalent concentrations. As expected, based on chemical properties and existing data, the silanes demonstrated toxicity in both systems with TMS being generally more toxic than TES. Overall, the results demonstrate that these *in vitro* test systems can provide valuable information relevant to predicting the likelihood of toxicity following inhalation exposure to chemicals in humans.

Regulatory agencies require information about the respiratory effects of substances that can be inhaled, and those data have historically been derived from tests on rats (OECD, 2009a,b, 2018a,b, 2019). However, there are anatomical and physiological differences between the rat and human respiratory tract that limit the precision with which the rat test predicts the human response. For example, rats are obligate nose breathers, whereas humans can breathe through either their nose or mouth (Harkema *et al.*, 2006), which can impact the deposition of an inhaled substance. In addition, anatomically, a rat’s nose has a more complex structure that better filters toxicants and protects the respiratory tract from inhaled substances. The monopodial branching pattern in rats’ airways allows a relatively unimpeded airflow whereas the bi- and tri-podial branching pattern in humans results in more turbulent air flow and therefore leads to more deposition (Hofmann and Asgharian, 2003). Additionally, there are other important differences between rats and humans, including ventilation rates and tidal volumes, cell and mucus composition, and metabolic and enzymatic activities (Green *et al.*, 2007; Travaglini *et al.*, 2020). To circumvent the limitations of animal tests, efforts have focused on optimizing human cell-based *in vitro* test systems that can efficiently and reliably predict the potential effects and mechanism of toxicity of inhaled substances on the human respiratory tract.

This study—the INSPIRE [*IN vitro* Systems to PredIct REspiratory toxicity] Initiative—was born out of a recommendation from a 2016 expert workshop on Alternative Approaches for Acute Inhalation Toxicity Testing to Address Global Regulatory and Non-Regulatory Data Requirements (Clippinger *et al.*, 2018a,b). The goal was to conduct a proof-of-concept case study to better understand the utility of cell-based test systems in predicting the effects of inhaled substances on the human respiratory tract. Extensive discussions with experts informed the choice of test chemicals (silanes) and biological systems used.

Silanes are chemicals that are used as reducing and coupling agents with applications in surface modifications (PubChem; Johnson *et al.*, 2016). Two chemicals belonging to the organic silane family (silicon esters)—triethoxysilane (TES; Chemical Abstracts Service [CAS] number: 998-30-1, HSi(OCH_2_CH_3_)_3_, Figure 1A) and trimethoxysilane (TMS; CAS number: 2487-90-3, HSi(OCH_3_)_3_, Figure 1B)—were assessed in this study (NRC, 2012a). These silanes are used as precursors to complex functional silanes and become a vapor in the production line. They are handled in closed systems so occupational exposure is unlikely but may occur in case of an accident or if personal protective equipment recommendations (eg, respirator and protective clothing) are not followed.

**Figure 1.**
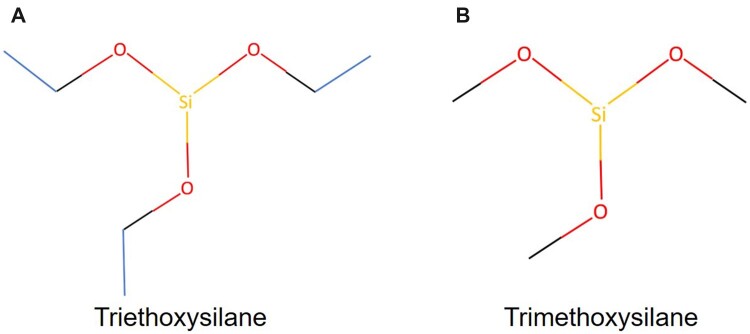
Chemical structure of triethoxysilane (TES, A) and trimethoxysilane (TMS, B).

In contact with water, these silanes react within a few seconds to minutes to produce monosilicic acid (Si(OH)_4_), and ethanol (CH_3_CH_2_OH, in the case of TES) or methanol (CH_3_OH, in the case of TMS). Therefore, any potential exposure would be expected to both the parent substance and the hydrolysis products. Although both silanes are acutely toxic when inhaled and are classified as skin and eye irritants (see Table 1 and Supplementary Table 1), toxicity of their hydrolysis products is low, with no known inhalation toxicity of monosilicic acid (noncrystalline) and 6 h inhalation lethal concentration 50 of ethanol and methanol reported as >50 mg/l and 87.5 mg/l, respectively (the European Chemicals Agency [ECHA] registered dossiers).

**Table 1.** Physical and chemical properties of triethoxysilane and trimethoxysilane (information obtained from ECHA registered dossiers)

Parameter	Triethoxysilane	Trimethoxysilane
Synonyms	TES, triethoxysilicon	TMS
Globally harmonized system (GHS) category	Inhalation GHS category 2 (OECD Test Guideline [TG] 403; in rats) Skin irritation GHS category 2 (OECD TG 404; in rabbits) Eye irritation GHS category 1 (OECD TG 405; in rabbits)	Inhalation GHS category 1 (OECD TG 403; in rats) Skin corrosion GHS category 1 (OECD TG 404; in rabbits) Eye irritation GHS category 2 (OECD TG 405; in rabbits)
CAS registry no.	998-30-1	2487-90-3
Chemical formula	HSi(OCH_2_CH_3_)_3_	HSi(OCH_3_)_3_
Molar mass	163.27	122.22
Physical state	Colorless liquid	Colorless liquid
Odor	Ester odor	Ester odor
Melting point	−170°C	−114°C
Boiling point	131°C	84.4°C
Flash point	26°C (closed cup)	7.2°C (closed cup)
Solubility (in water)	Reacts with water liberating ethanol	Reacts with water liberating methanol
Vapor pressure	8.5 mmHg at 25°C	76 mmHg at 25°C
Density	0.89 g/cm^3^ at 25°C	0.86 g/cm^3^ at 20°C
Half-life for hydrolysis	60 min at 20°C–25°C	17 s at 2°C
Hydrolysis products	Monosilicic acid (Si(OH)_4_) and ethanol (CH_3_CH_2_OH)	Si(OH)_4_ and methanol (CH_3_OH)

Due to rapid hydrolysis, silanes are unsuitable for testing in submerged *in vitro* systems. Therefore, under the INSPIRE Initiative, 2 cell-based systems were exposed to silanes at the air-liquid interface to assess whether their toxicity can be ranked using *in vitro* models. Because silanes cause damage throughout the respiratory tract, including to the bronchi (Supplementary Table 1, PubChem), a human bronchial epithelial cell line (BEAS-2B) and a reconstructed human bronchial tissue model (MucilAir) were selected for this study. Models based on human cells and that capture mechanisms of inhalation toxicity were used to maximize human relevance. With a goal to aid future *in vitro* testing, this study also shares lessons learned.

## Materials and methods

The manufacturer information and catalog details of the chemicals and the equipment used in this study can be found in Table 2.

**Table 2.** Materials used in this study and their source/manufacturer

Product Name	Source/Manufacturer	Catalog No.
BEAS-2B cell line	American Type Culture Collection (ATCC), Manassas, Virginia	CRL-9609
BEBM Bronchial Epithelial Cell Growth Basal Medium	Lonza, Basel, Switzerland	CC-3171
BEGM Bronchial Epithelial Cell Growth Medium BulletKit	Lonza, Basel, Switzerland	CC-3170
Bovine serum albumin	Sigma-Aldrich, Saint Louis, Missouri	A9647
Cell culture flask, 250 ml, 75 cm², PS	Greiner Bio-One nv, Vilvoorde, Belgium	658170
Cell culture multiwell plate, 6 well, PS, clear	Greiner Bio-One nv, Vilvoorde, Belgium	657160
Collison-type atomizer	Topas, Dresden, Germany	ATM-220
Corning Transwell polyester membrane cell culture inserts, 6-well format	Sigma-Aldrich, St Louis, Missouri	CLS3450
Cytotoxicity LDH Assay Kit-WST	Tebu-bio, Boechout, Belgium	Ck12-05
CytoTox-ONE Homogeneous Membrane Integrity Assay	Promega, Madison, Wisconsin	G7891
Formaldehyde	Sigma-Aldrich, St Louis, Missouri	8775
Fibronectin human plasma	Sigma-Aldrich, St Louis, Missouri	F2006
Gibco DPBS, calcium, magnesium	ThermoFisher Scientific, Waltham, Massachusetts	11580456
Isoproterenol hydrochloride	Merck KGaA, Darmstadt, Germany	1351005
Lipopolysaccharides from *Escherichia coli* O111: B4	Sigma-Aldrich, St Louis, Missouri	L4391
Mass flow controllers	Bronkhorst, Veenendaal, The Netherlands	Low flow ΔP/EL-flow
Microplate, 96 well, PS, F-bottom (chimney well)	Greiner Bio-One nv, Vilvoorde, Belgium	655094
Millicell ERS-2 voltammeter and electrodes	Merck KGaA, Darmstadt, Germany	MERS00002, MERSSTX01
MilliQ water	Merck KGaA, Darmstadt, Germany	Not available
Molecular Probes PrestoBlue Cell Viability Reagent	ThermoFisher Scientific, Waltham, Massachusetts	12023725
MucilAir	Epithelix Sàrl, Geneva, Switzerland	EP01MD
MucilAir Medium	Epithelix Sàrl, Geneva, Switzerland	EP04MM
Multimode microplate reader CLARIOstar Plus	BMG Labtech, Ortenberg, Germany	Not available
Nitrogen	Linde (Cryogenic tank), The Netherlands	2420901
Nitrogen dioxide	Air Liquide (Cylinder), The Netherlands	Not available
Paraformaldehyde 16%	Electron Microscopy Sciences, Hatfield, Pennsylvania	15710
Polyvinylpyrrolidone	Sigma-Aldrich, St Louis, Missouri	PVP40
PureCol	Advanced BioMatrix, Carlsbad, California	5005
Sisson-Ammons Video Analysis (SAVA) software and basler acA1300–200 µm USB3 video camera	Ammons Engineering, Clio, Michigan	Not available
Sodium chloride	Sigma-Aldrich, St Louis, Missouri	S7653
Total hydrocarbon analyzer	JUM Engineering GmbH, Karlsfeld, Germany	3-300
Triethoxysilane	Fisher Scientific S.P.R.L., Brand Alfa Aesor, Merelbeke, Belgium	11449567
Trimethoxysilane	Fisher Scientific S.P.R.L., brand Acros Organics, Merelbeke, Belgium	10014293
Trypsin/EDTA, 1X	LGC, Teddington, UK	ATCC-30-2101
V-PLEX Custom Human Biomarkers Proinflammatory Panel1 (human): Human IL-2, Human IL-6, Human IL-8, and Human TNF-α	Meso Scale Diagnostics, Rockville, Maryland	K151A9H-2
VITROCELL 6/4 CF Stainless Steel Exposure Module	VITROCELL Systems, Waldkirch, Germany	Not available
Zeiss Axiovert 10 microscope	Zeiss, Jena, Germany	Not available

###  

####  

##### Experimental design

Three test concentrations (low, mid, and high) for both chemicals were selected for the main study based on cytotoxicity (lactate dehydrogenase [LDH] release) in range-finding experiments (data not shown). Table 3 provides the number of technical replicates for both systems used in this study. For BEAS-2B cells, at least 3 biologically independent runs were performed (*N* = 3). For BEAS-2B only, TES and TMS exposures were not always run on the same day. Therefore, some controls were performed up to 5 times (*N* = 5). For MucilAir tissues, 5 experimental runs (*N* = 5) were performed (3 randomly selected donors, 1 donor tested 3 times; see Table 4 for donor information provided by the supplier). For this study, inter- and intra-donor variability was not assessed.

**Table 3.** Number of technical replicates used per cellular effect and post-exposure time point for BEAS-2B cells and MucilAir

Cellular Effect	Biological Test System			
	BEAS-2B	MucilAir		
*Post-exposure time point*	*19–24 h*	*−24 h*	*19–24 h^a^*	*7 days*
TEER	NA	4	3	2
CBF	NA	4	3	2
Cell viability	3	NA	1	2
Cytotoxicity	3	NA	3	2
Secreted inflammatory markers	3	NA	3	2
Histology	NA	NA	1	2

Abbreviation: NA, not assessed.

a One additional technical replicate was used for experimental run 5 for each cellular effect.

**Table 4.** Donor information provided for MucilAir tissues

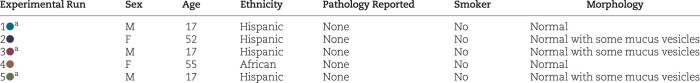

Abbreviations: F, female; M, male.

a Donor is the same for experimental runs 1, 3, and 5.

All exposures were carried out by generating silane vapors and diluting them with nonhumidified nitrogen (N_2_) gas (referred to as silane vapors in this manuscript). A 30-min exposure duration was selected based on preliminary results from this study showing a 60-min exposure caused an approximately 20% decrease in viability of BEAS-2B cells (data not shown), as also observed in similar studies with aerosol exposures (Mistry *et al.*, 2020; Rossner *et al.*, 2019). Samples were collected after 19–24 h for BEAS-2Bs and MucilAir and after 7 days for MucilAir. A range of 19–24 h is provided because exposures were done consecutively over the course of 5 h but all samples were harvested at the same time on the next day to guarantee same day processing. The following exposure conditions and controls were included in all experimental runs (*n* = 3 each, except *n* = 4 for MucilAir experimental run 5):

- Exposure to TES at 6.42, 320.77, or 962.30 mg/m^3^ (1, 50, 150 ppm) for BEAS-2B and 481.15, 962.30, or 1924.60 mg/m^3^ (75, 150, and 300 ppm) for MucilAir.
- Exposure to TMS at 4.80, 120.06, or 408.20 mg/m^3^ (1, 25, and 85 ppm) for BEAS-2B and 120.06, 480.24, or 1440.71 mg/m^3^ (25, 100, and 300 ppm) for MucilAir.
- An unexposed incubator control (IC, 37°C and 5% CO_2_) to account for any artifacts resulting from the exposure system.
- Sodium chloride (NaCl; 0.9%) exposure as an additional control to account for effects due to dry exposure conditions.
- Exposure to dry, nonhumidified N_2_ because TES and TMS were diluted in N_2_ gas (sham exposure).
- Exposure to nitrogen dioxide (NO_2_ at 45.19 mg/m^3^ [25 ppm] for BEAS-2B and 1445.97 mg/m^3^ [800 ppm] for MucilAir) as a positive control as NO_2_ has been previously shown to elicit toxicity in the bronchioles and alveolar region (Foster *et al.*, 1985; NRC, 2012b; Poynter *et al.*, 2006).
- Incubation with lipopolysaccharide (LPS; 20 μg/ml for BEAS-2B and 200 μg/ml for MucilAir) as an assay-specific positive control of inflammatory stimulation. In the case of MucilAir, 1 tissue was treated with LPS for the 19–24 h time point.
- Incubation with isoproterenol hydrochloride (ISO; 50 µM) as a positive control increasing cilia beating frequency (CBF). One MucilAir tissue was treated with ISO and assessed for changes in CBF at the 19–24 h time point.
- Incubation with lysis buffer (provided with the assay kit) as a positive control for LDH release assay.

##### Biological test systems

The normal human bronchial epithelial BEAS-2B cell line was obtained from the American Type Culture Collection (ATCC, Manassas, Virginia). The BEAS-2B cell line was established in 1988 by immortalizing nontumorigenic human bronchial cells using an adenovirus 12-SV40 hybrid virus, and it is commonly used in pulmonary (PU) research (Reddel *et al.*, 1988). BEAS-2B cells express proteins typical for epithelial cells, such as cytokeratins 8 and 18 and E-cadherin (Han *et al.*, 2020). According to Oesch *et al.*, BEAS-2B cells showed high overall similarity in gene expression of metabolizing enzymes compared with fresh human bronchial tissue (Oesch *et al.*, 2019). The thickness of a monolayer of BEAS-2B cells is 5–10 µm. BEAS-2B cells fail to form a tight barrier (low transepithelial electrical resistance; TEER), mucin production is low, and they express surface markers typical for mesenchymal stem cells (Bhowmick and Gappa-Fahlenkamp, 2016; Garcia-Canton *et al.*, 2013; Han *et al.*, 2020; Rossner *et al.*, 2019).

For this study, BEAS-2B cells were grown in T-75 culture flasks (Greiner Bio-One nv, Vilvoorde, Belgium) precoated with 15 ml of 10 µg/ml human plasma fibronectin (2 µg/cm^2^, Sigma-Aldrich, St Louis, Missouri), 30 µg/ml PureCol (6 µg/cm^2^, Advanced BioMatrix, Carlsbad, California), and 10 µg/ml bovine serum albumin (2 µg/cm^2^, Sigma-Aldrich, St Louis, Missouri) dissolved in bronchial epithelial cell basal medium (BEBM, Lonza, Basel, Switzerland). The bronchial epithelial cell growth medium (BEGM) consisted of BEBM supplemented with all ingredients of the BEGM BulletKit (Lonza, Basel, Switzerland) except bovine pituitary extract (BPE) because preliminary experiments showed that BPE is not needed to culture BEAS-2B cells. The cells were kept in a humidified atmosphere at 37°C and 5% carbon dioxide (CO_2_). Before reaching 80% confluence, the cells were subcultured using 0.25% Trypsin/0.53 mM EDTA solution (LGC, Teddington, UK) containing 0.5% polyvinylpyrrolidone (Sigma-Aldrich, St Louis, Missouri). The medium was refreshed every 2–3 days and cells were subcultured every 4–5 days (1500–3000 cells/cm^2^). Cells were used until passage 20.

MucilAir is a fully differentiated human airway epithelium tissue model that consists of primary human nasal or bronchial epithelial cells isolated from biopsies (Huang *et al.*, 2009) (Epithelix Sàrl, Geneva, Switzerland). The thickness of MucilAir tissues is about 50 µm. The cells are differentiated at the air-liquid interface and form a pseudostratified epithelial layer, which is typical for the conducting airways. MucilAir has been used to test many chemicals and to assess the production of mucus, active ion transport, barrier integrity, metabolic activity (eg, CYPs), secretion of cytokines, chemokines, and metalloproteinases. MucilAir maintains a normal phenotype for a long period of time, allowing for repeated exposures (Baxter *et al.*, 2015; Sauer *et al.*, 2013)_._ For this study, healthy MucilAir tissues from bronchial region were obtained from 3 individual and randomly selected nonsmoking donors, 1 male and 2 females (see Table 4 for donor information). Upon receiving MucilAir tissues, the inserts were placed in an incubator (37°C, 5% CO_2_) for 1 h after which they were removed from the nutritive gel (proprietary) and transferred to a new 24-well plate filled with 700 µl warm MucilAir Medium (Epithelix Sàrl) at 37°C. The plate was put in the incubator for normal culturing (37°C, 5% CO_2_). After 72 h, the basal medium was changed (700 µl) and each MucilAir insert was washed apically with MucilAir Medium to remove mucus, minimizing the risk of interference with the experiments. For this, 200 µl of warm medium at 37°C was added for 10–20 min on the apical surface of the inserts and incubated at 37°C. After incubation, 100 µl of apical medium was pipetted up and down 3 times to detach the mucus from the surface, after which all apical medium was gently removed by aspiration. As a part of the washing step, basal medium was also changed. After 48 h (1 day before exposure), the washing step was repeated to remove mucus. In addition, TEER and CBF were measured after washing (see section on CBF and TEER determination). The inserts were placed in the incubator for another 24 h until exposure.

##### Generation and characterization of test atmospheres

**Figure 2.**
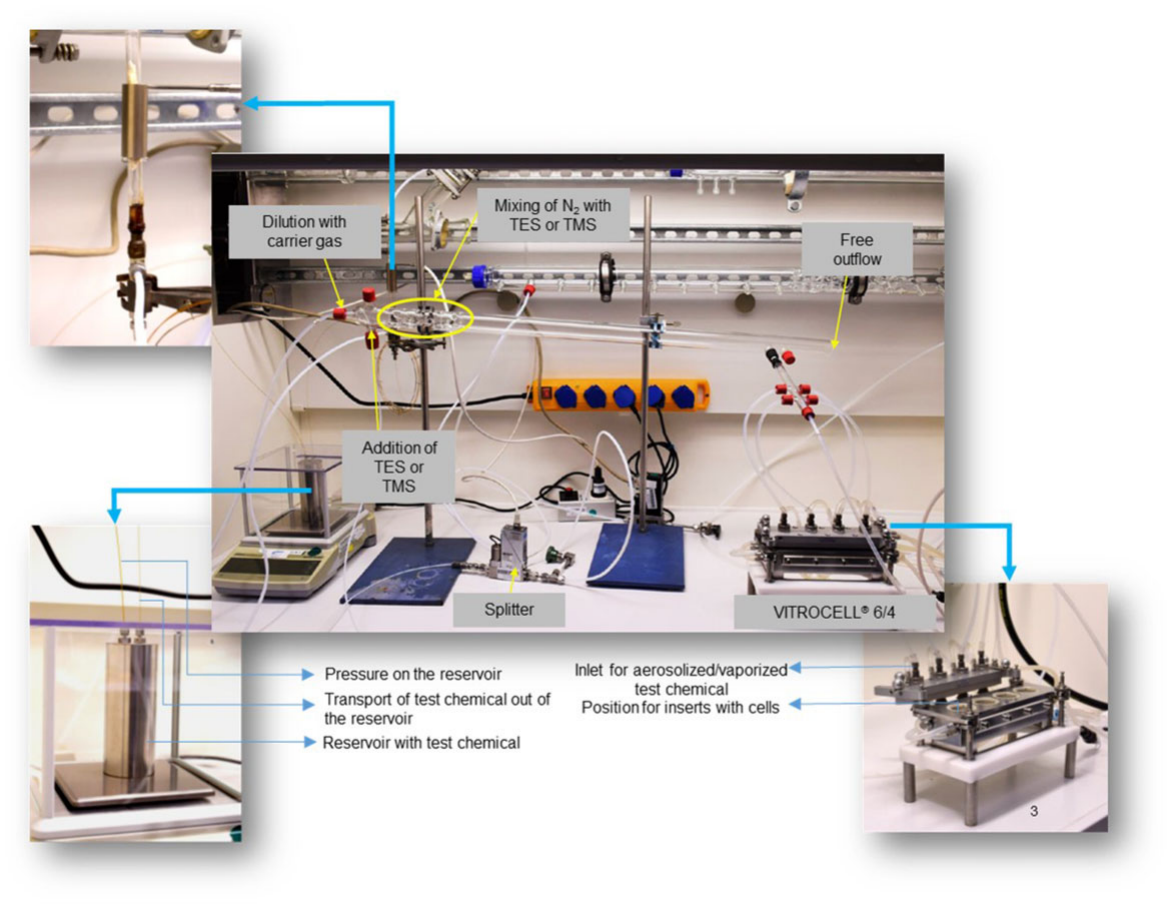
Vapor generation and cell exposure set up of silanes.

Silane vapors were generated using a capillary dosage system (Goelen *et al.*, 1992) and diluted in dry N_2_ (Linde, The Netherlands). This technique, where a fluid is injected through a capillary, thermally heated, evaporated, and injected dynamically into a gas stream, is theoretically based on Poiseuille’s equation, which allows the calculation of a laminar flow through a cylindrical tube. By altering the radius or pressure difference over the tube, a broad concentration range can be generated. A splitter mass flow controller (MFC, Bronkhorst, Veenendaal, The Netherlands) was installed to reach the lower concentrations. This MFC only takes a part of the primary flow through the capillary (splits it) and transfers this part to the glass distribution line. Briefly, a reservoir was filled with the silane and placed on an analytical balance. Pressure was set onto the closed recipient by use of a second capillary, and the outgoing liquid emerged at the outlet of the capillary where it was retained by a cotton plug and evaporated by a local heating element. The primary N_2_ gas flow transferred the silane vapors to the glass distribution line. Figure 2 shows the generation and cell exposure set up of silanes. The stability of the silanes in the carrier gas was monitored by the online instrument total hydrocarbon (THC) analyzer (Model 3-300, JUM Engineering GmbH, Karlsfeld, Germany). The generated concentrations were determined by using the measured value of the THC analyzer. The THC analyzer was calibrated whenever an experiment was performed (set to zero using N_2_ and consequently spanned with propane). A known concentration of silanes was offered earlier (separately) to the THC monitor, so a response factor could be calculated. This conversion factor was then used during the experiments to convert the monitor value to concentrations of TES and TMS.

For NO_2_ (Air Liquide, The Netherlands) exposure, which was used as a positive control, a gas bottle with diluted NO_2_ (1760.47 mg/m^3^, 974 ppm) was used. The use of MFCs connected to the gas bottle ensured stable gas flow and stable concentrations (45.19 mg/m^3^ [25 ppm] for BEAS-2B and 1445.97 mg/m^3^ [800 ppm] for MucilAir) of the compound in the test gas.

NaCl (Sigma-Aldrich, St Louis, Missouri) aerosols were generated using a Collison-type atomizer (Topas, Dresden, Germany) containing a 50 ml 0.9% NaCl suspension (in filter sterilized [0.22 µM] MilliQ water [Merck KGaA, Darmstadt, Germany, Darmstadt, Germany]) and N_2_ (5 bar). The NaCl-containing N_2_ flow leaving the atomizer (at 4 liters per minute [lpm]) was dried using a diffusion dryer and connected to the distribution unit. Liquid N_2_ was decompressed and fed to MFCs, through pressure reducers, to control the flow of N_2_ gas through the set up.

##### Exposure of BEAS-2B cells to test chemicals

BEAS-2B cells were seeded at a density of 50 000 cells/cm^2^ on Corning Transwell polyester membrane 6-well cell culture inserts with pore size of 0.4 µm (Sigma-Aldrich, St Louis, Missouri). The cell culture inserts were coated with the same coating solution and concentration as described earlier. The inserts seeded with cells were placed in a sterile 6-well plate, and BEGM was added to both sides, 2 ml basolateral and 1 ml apical. The plates were incubated for 48 h (37°C, 5% CO_2_). Immediately before exposure, cells were checked using a conventional inverted microscope, after which the BEGM was completely removed from the apical and basolateral side of all the inserts and 1 ml of fresh BEBM (without growth factors to stop the cells from growing further) was added basolaterally.

For exposure, inserts were transferred to a VITROCELL 6/4 exposure module (VITROCELL Systems, Waldkirch, Germany). The module was maintained at 37°C using a circulating water bath and each well was filled with 5.5 ml BEBM, allowing cells to be nourished from the basolateral side while being exposed at the air-liquid interface from the apical side. The distance between the inlet and insert (ie, trumpet height) was 3 mm and the flow of the test atmospheres at the apical surface of the cells was generated using a vacuum pump at a constant rate of 3.0 ml/min. All exposures were carried out for 30 min. Exposure concentrations are described in the Experimental design section.

After exposure, inserts were placed in a new, sterile 6-well plate with 1 ml fresh BEBM added basolaterally. The cells were not washed apically after exposure. After 19–24 h in the incubator, half of the basolateral medium (500 µl) from the inserts was collected for assessment of cytotoxicity and the other half was frozen and stored at −80°C for later assessment of cytokine secretion. The cells were used for the cell viability assay. At least 3 biologically independent experimental runs, using different cell passages, were performed for each exposure condition. After exposure, the VITROCELL exposure module and all tubings were thoroughly rinsed with 70% ethanol.

##### Exposure of bronchial MucilAir tissues to test chemicals

Each tissue insert was inspected under a conventional inverted microscope to ensure the quality of the epithelia. Tissues were transferred to a VITROCELL 6/4 exposure module with adaptors for 24-well inserts. The exposure module was maintained at 37°C using a circulating water bath and each well was filled with 5 ml MucilAir medium, allowing cells to be nourished from the basolateral side while being exposed at the air-liquid interface from the apical side. The distance between the inlet and insert (ie, trumpet height) was 3 mm and the flow of the test atmospheres at the apical surface of the cells was generated using a vacuum pump at a constant rate of 3.0 ml/min. All exposures were carried out for 30 min. Exposure concentrations are described in the Experimental design section.

After exposure, inserts were placed in a new, sterile 24-well plate with 500 µl MucilAir medium added basolaterally. The tissues were not apically washed directly after exposure. After 19–24 h of incubation, 200 μl of the basolateral medium was sampled for LDH assay and the remaining medium was stored at −80°C for cytokine measurements later. Fresh medium was added basolaterally. Next, TEER and CBF were measured after apical washing. One of the tissues was used for PrestoBlue assay and afterward fixed for histology (2 tissues for experimental run 5). The 2 remaining tissues were kept for another 6 days (until 7 days post-exposure) and medium was changed at 3 and 6 days post exposure. After 7 days post-exposure, basolateral medium was collected and sampled for LDH and the remaining medium was stored at −80°C for cytokine measurements. TEER and CBF were again measured. The tissues were then used for PrestoBlue assay and afterward fixed for histology. Five biologically independent runs, using different donors (see Table 4 for donor information), were performed for each exposure condition. After exposure, the VITROCELL exposure module and all tubings were thoroughly rinsed with 70% ethanol.

##### Conversion of exposure concentration from ppm to mg/m^3^

Because ppm depends on the molar mass of a substance, temperature, and pressure, comparison of results from 2 different substances or results obtained at different exposure conditions should be avoided. Therefore, all exposure concentrations and results were converted from ppm to mg/m^3^ according to the following equation:


$$\mathrm{mg}/m^{3}\;=\;\frac{\mathrm{ppm}\;\times \;m}{V_{m}},$$

where *m* is molar mass (g/mol) and *V*_m_ is the molar volume (*V*_m_ at 37°C is approximately 25.45 l/mol).

##### Measurement of mitochondrial activity to determine cell viability

Cell viability was analyzed 19–24 h (and additionally 7 days for MucilAir) post-exposure using the PrestoBlue Cell Viability Reagent (ThermoFisher Scientific, Waltham, Massachusetts). PrestoBlue assay indicates the metabolic competence of cells by measuring the reduction of resazurin to resorufin. When added to cells, the PrestoBlue reagent is modified by the reducing environment of viable cells and turns red in color and becoming highly fluorescent. This change can be detected using absorbance or fluorescence measurements. For this study, the manufacturer’s instructions were followed to assess cell viability. Briefly, after removing the basolateral medium, 1 ml of a 10% PrestoBlue solution in BEBM was added on the apical side for BEAS-2B cells or 200 µl of a 10% PrestoBlue solution in MucilAir Medium was added for MucilAir tissues. After incubation for 1 h at 37°C in the dark, fluorescence of 2 samples/well (90 µl each) from apical solution was measured in a white 96-well plate with clear bottom (Greiner, Bio-One nv) using a multimode microplate reader in fluorescence mode (excitation: 552 nm (22 nm), emission: 590 nm (20 nm), BMG Labtech, Ortenberg, Germany). Cell viability was expressed as the percentage of relative fluorescence intensity (RFI) of treated cells relative to the RFI of the N_2_ control cells.

##### Measurement of membrane integrity using lactate dehydrogenase assay to determine cytotoxicity

Two commercially available LDH detection kits were used in this study. For assessing cytotoxicity in BEAS-2B cells, the LDH detection kit CytoTox-ONE Homogeneous Membrane Integrity Assay (Promega, Madison, Wisconsin) was initially used (for runs 1 and 2 for TES and runs 1–3 for TMS-exposed BEAS-2B cells). Briefly, after exposure to test materials, the inserts with BEAS-2B cells were placed in a new, sterile 6-well plate containing 1 ml of BEBM, allowing cell recovery in a humidified incubator. After 19–24 h post-exposure, 2 technical replicates (100 μl each) from basolateral medium were used for the LDH assay per sample. The collected medium was incubated for 10 min with 100 μl LDH substrate mix in a 96-well plate. The reaction was stopped by the subsequent addition of 50 μl stop solution. As a positive control, 20 µl of lysis solution (included in the LDH assay kit) in 1 ml BEBM was added on the apical surface of one of the inserts and incubated for 19–24 h. Fresh BEBM incubated with LDH substrate mix was used as a background control. Fluorescence measurements were conducted using a multimode microplate reader (excitation wavelength of 560 nm and an emission wavelength of 590 nm, BMG, Labtech). However, due to issues encountered with the assay (eg, high fluorescence values for assay blanks), it was switched to LDH Assay Kit-WST according to Dojindo’s protocol (Tebu-bio, Boechout, Belgium) and used for BEAS-2B cells (run 3 for TES and TMS) and MucilAir tissues (all runs), per the manufacturer’s instructions. Briefly, after exposure, the inserts with BEAS-2B cells were placed in a new, sterile 6-well plate containing 1 ml of BEBM, allowing cell recovery in a humidified incubator. After 19–24 h post-exposure, 2 technical replicates of each 100 µl of the basolateral medium were used for the LDH assay per sample. The collected medium was incubated for 30 min with 100 μl LDH substrate mix in a 96-well plate. The reaction was stopped by the subsequent addition of 50 μl stop solution. As a positive control, 20 µl of lysis solution (included in the LDH assay kit) mixed with 1 ml of BEBM was added on the apical surface of one of the inserts and incubated for 19–24 h. Fresh BEBM incubated with LDH substrate mix was used as a background control. Absorbance measurements were conducted using a multimode microplate reader (wavelength: 490 nm, BMG, Labtech).

In MucilAir tissues, after 19–24 h and 7 days post-exposure, 2 technical replicates (100 μl each) from basolateral medium were used for the LDH assay per sample. The collected medium was incubated for 30 min with 100 μl LDH substrate mix in a 96-well plate. The reaction was stopped by the subsequent addition of 50 μl stop solution. As a positive control, 50 µl of lysis solution (included in the LDH assay kit) was added on the apical surface of 1 MucilAir tissue and incubated for 19–24 h. Fresh MucilAir Medium incubated with the LDH substrate mix was used as a background control. Absorbance measurements were conducted using a multimode microplate reader (wavelength: 490 nm, BMG, Labtech). Fold change in cytotoxicity levels relative to N_2_ exposure was calculated prior to statistical analysis.

##### Measurement of pro-inflammatory markers

For the measurement of pro-inflammatory markers (interleukin IL-2, IL-6, CXCL-8 [chemokine [C-X-C motif] ligand 8, also known as IL-8], and tumor necrosis factor-alpha [TNF-α] protein), the Meso Scale Discovery (MSD) V-PLEX Assay was performed according to the manufacturer’s protocol (Meso Scale Diagnostics, Rockville, Maryland). This assay is an electrochemiluminescent detection method and is a highly sensitive multiplex enzyme-linked immunosorbent assay. Calibration curves were used to calculate the cytokine concentrations, expressed in pg/ml. In preliminary experiments, a total of 10 inflammatory markers were assessed. IL-6 and CXCL-8 were the only ones with secretion levels of untreated samples consistently above the limit of detection and were the most sensitive markers upon exposure to the test chemicals. Therefore, only the results of IL-6 and CXCL-8 are shown.

At 19–24 h post-exposure, 500 µl of basolateral medium was collected in a 24-well plate and stored at −80°C until analysis using the MSD assay. As a positive control for inflammation, 1 insert of BEAS-2B cells was exposed to 20 µg/ml LPS on the apical side for 30 min after which the LPS was removed from the apical side without rinsing the cells. The basolateral medium was collected 19–24 h post-exposure. The MSD plate was washed 3 times using 150 µl of wash buffer (phosphate-buffered saline [PBS] 0.05% Tween-20, provided with the kit) per well. Buffer was removed by decanting between washes. Then, the calibrators were added, as well as the prepared samples, which were diluted 4-fold in assay diluent. The plate was sealed and incubated overnight at 4°C. Another washing step was performed 3 times, after which 25 µl of the detection antibody solution was added to each well. The plate was incubated at room temperature for 2 h on an orbital shaker (at 750 rpm), after which another wash step was performed in 3-fold. Afterward, 150 µl of the read buffer T was added to each well and the plate was analyzed on the MSD instrument. The same protocol was followed for samples obtained from MucilAir tissues, 19–24 h and 7 days post-exposure, except that only 200 µl of basolateral medium was collected and stored at −80°C until analysis.

The MSD software calculated the protein concentrations in each sample based on the calibrators that were incubated alongside the samples. Reported concentrations were then processed further via R (Bunn, 2008). Concentrations below the lower limit of detection (LLOD) were replaced by LLOD divided by 2. Subsequently, cytokine concentrations were normalized for cell viability (PrestoBlue) by dividing the cytokine concentration by the cell viability divided by 100. Significant changes in protein concentration were analyzed relative to N_2_ and were assessed as described in the Statistical analyses section.

##### H&E histology (MucilAir only)

A 4% paraformaldehyde solution (Electron Microscopy Sciences, Hatfield, Pennsylvania) in PBS with calcium and magnesium (PBS+; ThermoFisher Scientific, Waltham, Massachusetts) was prepared. The inserts were washed 3 times for 5 min in PBS+ and fixed for 30 min at room temperature using 4% paraformaldehyde solution (0.5 ml apical and 1 ml basolateral). After fixation, the inserts were washed 3 times for 5 min each in PBS+ in a new 24-well plate. After washing, the inserts were transferred to a 50-ml falcon tube filled with 50 ml PBS+ at 4°C and shipped to Histalim (France) for staining hematoxylin and eosin (H&E), embedding, sectioning, and imaging. For experimental runs 1–4, the membranes were excised out of the transwell inserts and transferred to an Eppendorf tube with PBS+ before shipping to Cerba for further processing. The stained tissues were digitized in bright field conditions using a NanoZoomer scanner (Hamamatsu Photonics). During shipping, several membranes got stuck on the lids of Eppendorf tubes and could not be used for histological analysis. Additionally, the duration for which the membranes were stored after fixing seemed to impact the sectioning of tissues for histology. Due to these issues, for run 5, the membranes were not cut out of the transwell inserts and were shipped for histology immediately after conclusion of the experimental run. Run 5 is the only experimental run with intact histological information.

##### Determination of cilia beating frequency and average active area (MucilAir only)

CBF was determined with the Sisson-Ammons Video Analysis (SAVA) software (Ammons Engineering, Clio, Michigan). On each insert, 2 microscopic fields were measured with a 10× objective on a Zeiss Axiovert microscope (Zeiss, Jena, Germany) with a Basler acA1300-200 µm USB3 video camera. A 5-second video (100 frames per second, 512 frames) was taken and further analyzed with the software. As a positive control, 1 tissue was exposed basolaterally for 1 h to 50 µM ISO. Significant changes in CBF and AAA compared with N_2_ for a particular time point (pre-, 19–24 h post-, or 7 days post-exposure) were assessed as described in the Statistical analyses section.

##### Determination of barrier integrity (TEER, MucilAir only)

Warm (37°C) MucilAir Medium (200 µl) was added on the apical surface of MucilAir tissues. The Millicell ERS-2 (Merck KGaA, Darmstadt, Germany) voltammeter was turned on and the electrode was washed in 70% ethanol and MucilAir Medium. The electrodes were equilibrated in the MucilAir medium for approximately 5 min. Care was taken to place the electrodes the same way for all inserts in order to decrease variability of the measurements. Electrical resistance was measured and the value was recorded in a laboratory book. The electrodes were not washed between measuring steps or between plates. After measuring all tissues, medium was gently aspirated from the apical surface of the MucilAir inserts. Care was taken not to damage the epithelium. Calculation to convert resistance to TEER: TEER (Ωcm^2^) = (resistance value [Ω] – 100 [Ω]) × 0.33 (cm^2^) with 100 Ω being the resistance of the membrane (blank) and 0.33 cm^2^ is epithelium’s surface. Significant changes in TEER compared with N_2_ for a particular time point (pre-, 19–24 h post-, or 7 days post-exposure) were assessed as described in the Statistical analyses section.

##### Proof-of-concept derivation of human equivalent concentration using *in vitro* data

As a proof of concept, the data generated in this study were used to calculate human equivalent concentrations (HEC). Benchmark dose modeling software (BMDS) version 3.2.0.1 was used to analyze the data of each cellular effect and derive the benchmark concentration (BMC) (U.S. EPA, 2022). U.S. EPA’s website (https://www.epa.gov/bmds) provides further information including user guides on how to use the BMDS. Briefly, the default settings for continuous model type were applied (ie, benchmark response [BMR] type being standard deviation; BMR factor of 1; confidence level of 0.95; normal distribution and constant variance). Absolute values were used to calculate BMC from data from pro-inflammatory markers, CBF, AAA, and TEER. Relative values (normalized to N_2_) were used for cell viability and cytotoxicity.

The best fitting model was selected based on the following: (1) the overall goodness-of-fit test *p*-value (high values are indicative of adequate fit), (2) the Akaike Information Criterion (AIC; smaller number indicates better fit), (3) scaled residuals (numbers greater than 2 or lower than −2 indicate questionable fit), and (4) the lower limit of the 95% confidence interval (BMCL; benchmark concentration lower-confidence limit). If models were comparable based on 1–3, the model with the lowest BMCL was chosen for reporting. Summary data tables of all models per cellular effect can be found in Supplementary Table 2A–V. The BMCL values were then used to calculate HECs as described next.

A modification of the regional gas dose ratio (RGDR) approach described in U.S. EPA (2012) was applied to derive HEC from the BMCL values for each cellular effect. The default dosimetric adjustment factor for portal-of-entry effects in the extrathoracic region of the respiratory tract is 1, whereas the RGDR for the tracheobronchial (TB) and PU regions is based on the animal-to-human ratio of the minute ventilation (*V*_E_) normalized to the surface area (SA) of the respiratory tract (ie, TB or PU) as depicted in the following equation:


$$\mathrm{RGDR}\;=\;(V_{E}/\mathrm{SA}{)}_{\mathrm{Animal}}÷(V_{E}/\mathrm{SA}{)}_{\mathrm{Human}}$$

The *V*_E_ of an adult human is 13 800 ml/min and the SA of the TB is 3200 cm^2^. To apply the *in vitro* data, the volume of air delivered per minute (ie, 3 ml/min) was used as a surrogate for *V*_E_ and the cell insert SA (ie, 4.6 cm^2^ for BEAS-2B and 0.3 cm^2^ for MucilAir) as a surrogate for SA. The SA of the TB region was used, based on the use of TB cells in the MucilAir tissue construct. Next, RGDR was multiplied with BMCL values to obtain HEC for a 30-min exposure. The HEC were further extrapolated from 30 min to 4 h by applying a duration-extrapolation multiplier of 0.125 (ie, 0.5 h/4 h) to the exposure concentration.

For simplicity, 37°C, 1 atmosphere, 100% of the inhaled chemical concentration reaching the bronchial region in humans, and 100% of the chemical depositing on and reacting with the cells (100% deposition efficiency) were assumed for these calculations. Because this calculation takes the SA of the *in vitro* system and the exposure duration into account, it may allow to better compare results obtained from different studies.

##### Statistical analysis

Significant changes were assessed by mixed models. A mixed model is a statistical model containing both the fixed effects and random effects. This is particularly relevant in the case of repeated measurements, which is represented here by the technical replicates within an experimental run. Hence, experimental run was considered as a random factor. R-packages for mixed model analyses “lme4” (Bates *et al.*, 2015) and “lmerTest” (Kuznetsova *et al.*, 2017) were applied. lme4 is used to fit the model, whereas lmerTest calculates the *p*-value of significance using a Type III ANOVA with Satterthwaite’s degrees of freedom method. *p*-value less than .05 was used as cut-off for statistical significance.

## Results for BEAS-2B cells

###  

#### Assessment of cell viability and cytotoxicity after silane exposure of BEAS-2B cells

**Figure 3.**
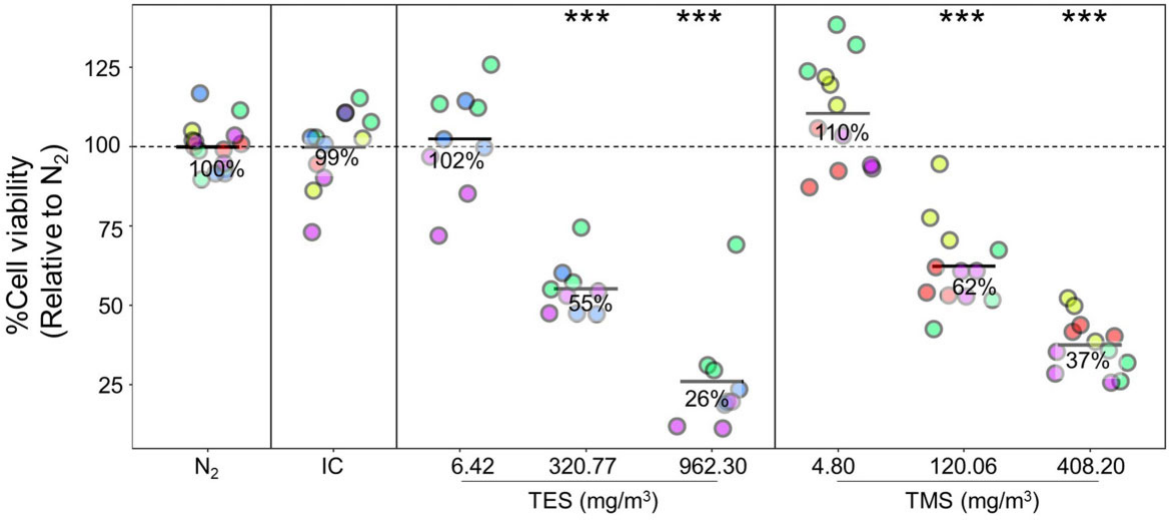
Assessment of cell viability in BEAS-2B cell line. BEAS-2B cells were exposed to controls (N_2_, IC, NaCl, and NO_2_) or TES (6.42, 320.77, or 962.30 mg/m^3^) or TMS (4.80, 120.06, or 408.20 mg/m^3^) vapor for 30 min at the air-liquid interface and cell viability was assessed using PrestoBlue assay after 19–24 h. The different colors represent different days of exposure (=experimental runs) with each run having 3 technical replicates (*n* = 3). The graphs show results from 3 separate experimental runs for TES (*N* = 3), 4 experimental runs for TMS (*N* = 4), and 5 experimental runs for the N_2_ control. Viability was normalized to the N_2_ control cells (=100% cell viability). Abbreviations: IC, incubator control; N_2_, nitrogen gas; NaCl, sodium chloride; TES, triethoxysilane; TMS, trimethoxysilane. The asterisk (*) shows statistical significance compared with N_2_ (negative control); *p <* *.05; **p <* *.01; ***p <* *.001.

BEAS-2B cells were exposed to 6.42, 320.77, or 962.30 mg/m^3^ TES and to 4.80, 120.06, or 408.20 mg/m^3^ TMS for 30 min. At 19–24 h post-exposure, cell viability was measured using the resazurin-based PrestoBlue assay and cytotoxicity was measured using the LDH detection assay. Three experimental runs were conducted for TES but 4 runs were performed for TMS because of issues with the LDH cytotoxicity assay. Each experimental run is shown in a different color representing experiments performed on different days and each dot represents 1 technical replicate. No significant changes were observed for the negative controls but exposure to the positive control, NO_2_, caused a significant decrease in cell viability relative to N_2_ (Supplementary Figure 1). A concentration-dependent decrease in cell viability was observed following TES and TMS exposure (Figure 3). The low concentration of TES and TMS showed no statistically significant change in cell viability compared with negative controls. Relative to the negative exposure control (N_2_) as well as the IC, the viability of TES-exposed cells was reduced to 55% at 320.77 mg/m^3^ (mid concentration) and to 26% at 962.30 mg/m^3^ (high concentration) (*p <* .001). Viability of TMS-exposed cells was reduced to 62% at 120.06 mg/m^3^ (mid concentration) and to 37% at 408.20 mg/m^3^ (high concentration) (*p < *.001), compared with the N_2_ negative control.

Cytotoxicity was assessed using a LDH assay. However, due to issues with the initial florescence-based kit used and switching to another absorbance-based LDH kit mid-study, there is only 1 experimental run using the new, optimized kit. Therefore, the results are only shown in Supplementary material. Despite these practical issues, a concentration-dependent increase in cytotoxicity was observed for both TES and TMS (Supplementary Figure 2).

#### Assessment of inflammatory marker secretion after silane exposure of BEAS-2B cells

At 19–24 h post-exposure, the basolateral medium was collected to assess the secretion of pro-inflammatory markers. From initially 10 markers tested in the concentration-range finding studies (data not shown), the selection was reduced to 4 markers: IL-2, IL-6, CXCL-8 (also known as IL-8), and TNF-α. All 4 inflammatory markers showed significant increases for cells treated with LPS (positive control) but basal IL-2 and TNF-α secretion levels were often below the level of detection and therefore not further analyzed (data not shown). Inflammatory mediators for the highest concentration were not assessed for either TES or TMS because of the significant decrease in cell viability observed (less than 50% of cells viable).

**Figure 4.**
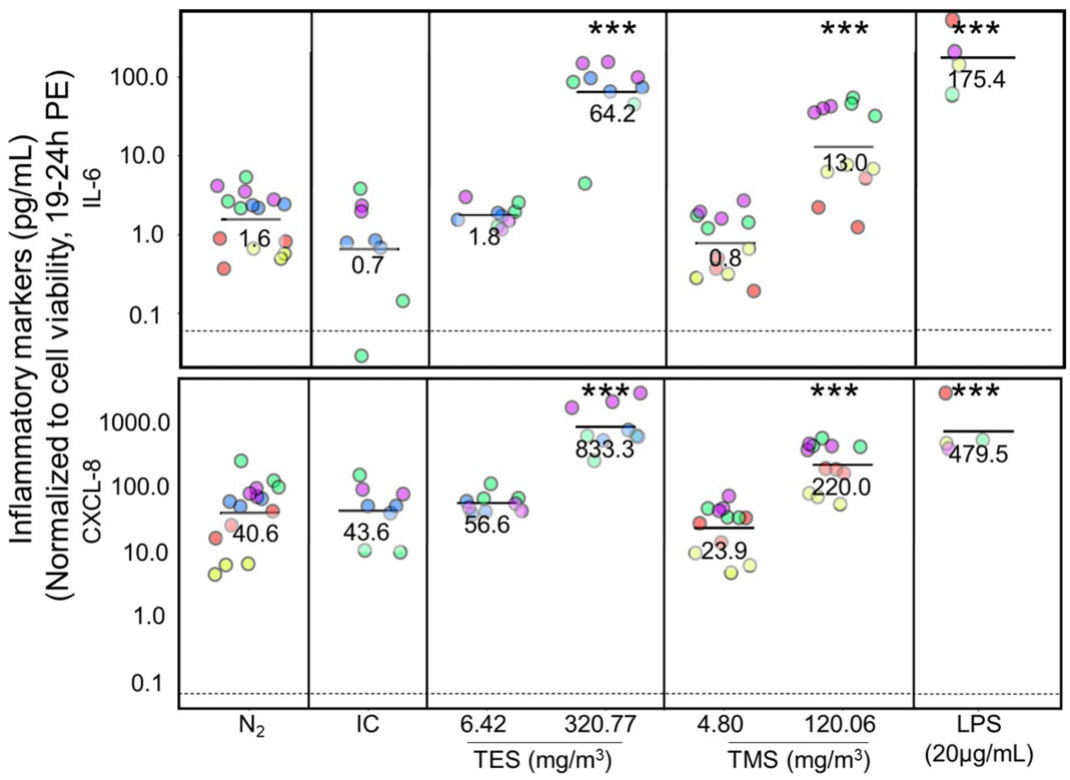
Assessment of inflammatory marker secretion in BEAS-2B cell line. BEAS-2B cells were exposed to controls (N_2_, IC, NaCl, or NO_2_) and test chemicals TES (6.42, 320.77, or 962.30 mg/m^3^, *N* = 3) or TMS (4.80, 120.06, or 408.20 mg/m^3^, *N* = 4) for 30 min at the air-liquid interface. Samples from the basolateral medium were collected for assessment of inflammatory markers after 19–24 h. Data represent exposure to TES at 6.42 or 320.77 mg/m^3^ and to TMS at 4.80 or 120.06 mg/m^3^; the highest concentrations were not included because of cell viability <50% observed at those concentrations (Figure 3). The different colors represent different days of exposure (=experimental runs) with each run having 3 technical replicates (*n* = 3). The graphs show results from 3 separate experimental runs for TES (*N* = 3), 4 experimental runs for TMS (*N* = 4), and 5 experimental runs (*N* = 5) for the N_2_ control. The data points show the secretion of IL-6 and CXCL-8 (IL-8) normalized to cell viability. *Y*-axis is shown in log-scale. Abbreviations: IC, incubator control; IL, interleukin; N_2_, nitrogen gas; NaCl, sodium chloride; TES, triethoxysilane; TMS, trimethoxysilane. The asterisk (*) shows statistical significance compared with N_2_ (negative control); *p <* *.05; **p <* *.01; ***p <* *.001.

The secretion of IL-6 and CXCL-8, 2 widely used pro-inflammatory mediators to assess potential toxicity of inhaled substances (Balharry *et al.*, 2008; Cesta *et al.*, 2021; Dankers *et al.*, 2018; El-Shimy *et al.*, 2014; Sivars *et al.*, 2018; Welch *et al.*, 2021), was significantly altered. Secretion of IL-6 and CXCL-8 was similar for all negative controls (1.6 and 40.6 pg/ml for N_2_, 0.7 and 43.6 pg/ml for IC, and 2.8 and 46.0 pg/ml for NaCl, see Supplementary Figure 3). The lowest exposure concentration of 6.42 mg/m^3^ TES and 4.80 mg/m^3^ TMS did not alter the secretion of IL-6 and CXCL-8 (1.8 and 56.6 pg/ml for TES, and 0.8 and 23.9 pg/ml for TMS, respectively; Figure 4). However, the secretion was significantly elevated for the mid concentration (64.2 pg IL-6/ml and 833.3 pg CXCL-8/ml for 320.77 mg/m^3^ TES, and 13.0 pg IL-6/ml and 220.0 pg CXCL-8/ml for 120.06 mg/m^3^ TMS) (Figure 4). For the mid concentration of TES, IL-6 secretion increased 40-fold and CXCL-8 secretion increased 20-fold relative to N_2_ control. For the mid concentration of TMS-treated tissues, compared with N_2_, a 8-fold increase in IL-6 and a 5-fold increase in CXCL-8 was observed.

## Results for bronchial MucilAir tissues

The reconstructed human bronchial tissue model, MucilAir, was exposed in a manner similar to the BEAS-2B cells. The tissues were exposed to 481.15, 962.30, or 1924.60 mg/m^3^ TES and 120.06, 480.24, or 1440.71 mg/m^3^ TMS for 30 min. In addition to the cellular effects tested in BEAS-2B cells, CBF and average active area (AAA), and barrier integrity (measured using TEER) were assessed in MucilAir tissues. A 7 day recovery period was also added. For each experimental run, with the exception of run 5 (where 2 technical replicates each were assessed), 1 technical replicate per experimental run was used for the 19–24 h time point, and 2 replicates were used at 7 days post exposure (see Table 3 for the number of replicates). The same controls were used as for BEAS-2B cells. Some intra- (within the same donor) and inter-donor (between donors) variability was observed but not further analyzed as the goal of this study was to assess whether conclusive results can be obtained with randomly selected donors.

###  

#### Assessment of cell viability and cytotoxicity in bronchial MucilAir tissues

**Figure 5.**
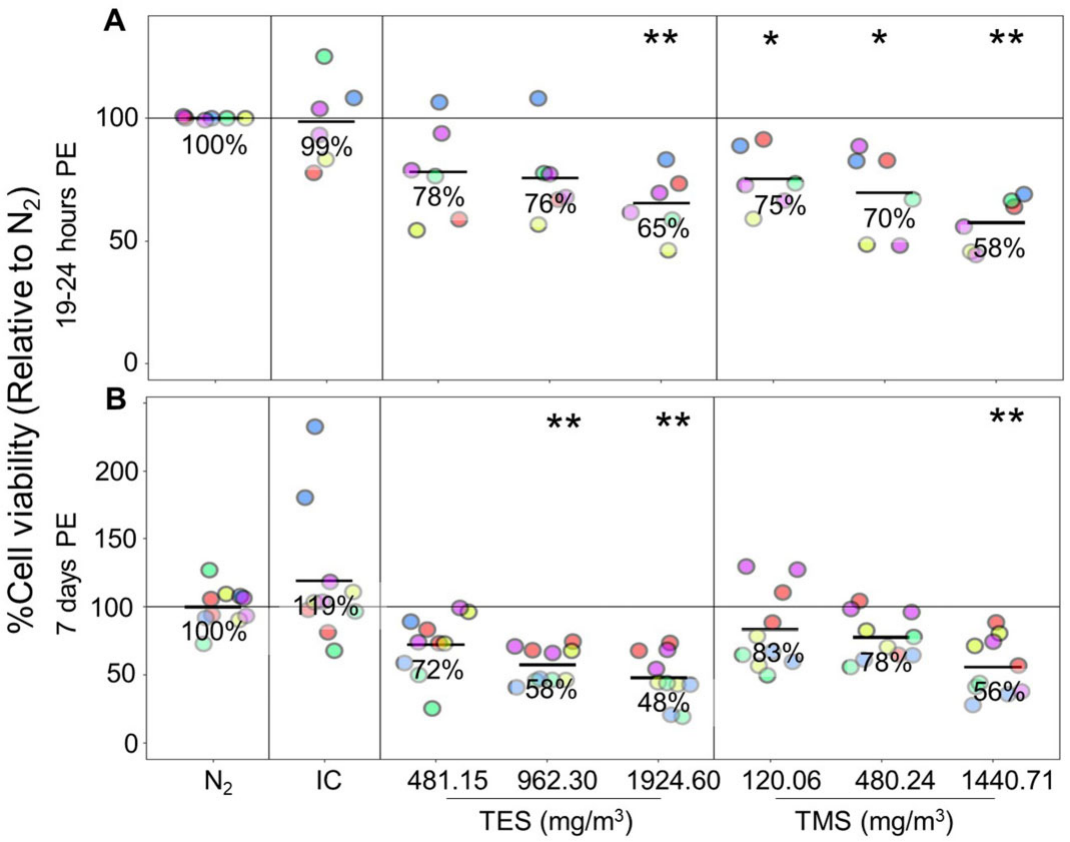
Assessment of cell viability in bronchial MucilAir tissues. MucilAir tissues were exposed to 481.15, 962.30, or 1924.60 mg/m^3^ TES and 120.06, 480.24, or 1440.71 mg/m^3^ TMS for 30 min at the air-liquid interface. Cell viability was assessed using PrestoBlue assay after 19–24 h or 7 days. Cell viability was expressed as the percentage of the fluorescence of treated cells relative to the fluorescence of the N_2_ control cells. (A) and (B) show viability of cells treated with TES and TMS relative to N_2_ control cells after 19–24 h and 7 days, respectively. Abbreviations: IC, incubator control; N_2_, nitrogen gas; PB, PrestoBlue; PE, post-exposure; TES, triethoxysilane; TMS, trimethoxysilane. (*n* = 5, the asterisk [*] shows statistical significance compared with N_2_ [negative control]; *p <* *.05; **p <* *.01; ***p <* *.001).

MucilAir tissues were exposed at the air-liquid interface to TES and TMS vapor for 30 min, and cell viability was assessed using the resazurin-based PrestoBlue assay at 19–24 h and 7 days post-exposure. Because resazurin has been reported to be a respiratory irritant, the tissues were fixed for histology after the PrestoBlue assay and not used for further analyses. Because only 1 technical replicate was used to measure cell viability after 19–24 h post-exposure, the results need to be interpreted with caution, but the data suggest that there may be a concentration-dependent decrease in cell viability (Figure 5). No significant changes were observed for the negative controls but exposure to the positive control, NO_2_, caused a significant decrease in cell viability relative to N_2_ (Supplementary Figure 4). At 19–24 h after exposure, tissue viability decreased to 78% (not significant [ns]), 76% (ns), and 65% (*p* < .01) for TES, and to 75% (*p* < .05), 70% (*p* < .05%), 58% (*p* < .01%) for TMS for low, mid, and high concentrations compared with N_2_ exposed tissues (Figure 5A). At 7 days post-exposure, cell viability relative to negative controls was significantly decreased for mid (58%, *p* < .01) and high (48%, *p* < .01) concentrations of TES, and only for the high (56%, *p* < .01) concentration of TMS indicating that both silanes damaged the tissues substantially (Figure 5B). Interestingly, although experimental runs 1 (red), 3 (green), and 5 (purple) were tissues originating from the same donor, the results do not cluster around the same data points.

Similar to results from BEAS-2B cells, a concentration-dependent increase in cytotoxicity (LDH assay) was also observed in the case of MucilAir tissues for both TES and TMS (Supplementary Figure 5).

#### Assessment of inflammatory marker secretion in bronchial MucilAir tissues

**Figure 6.**
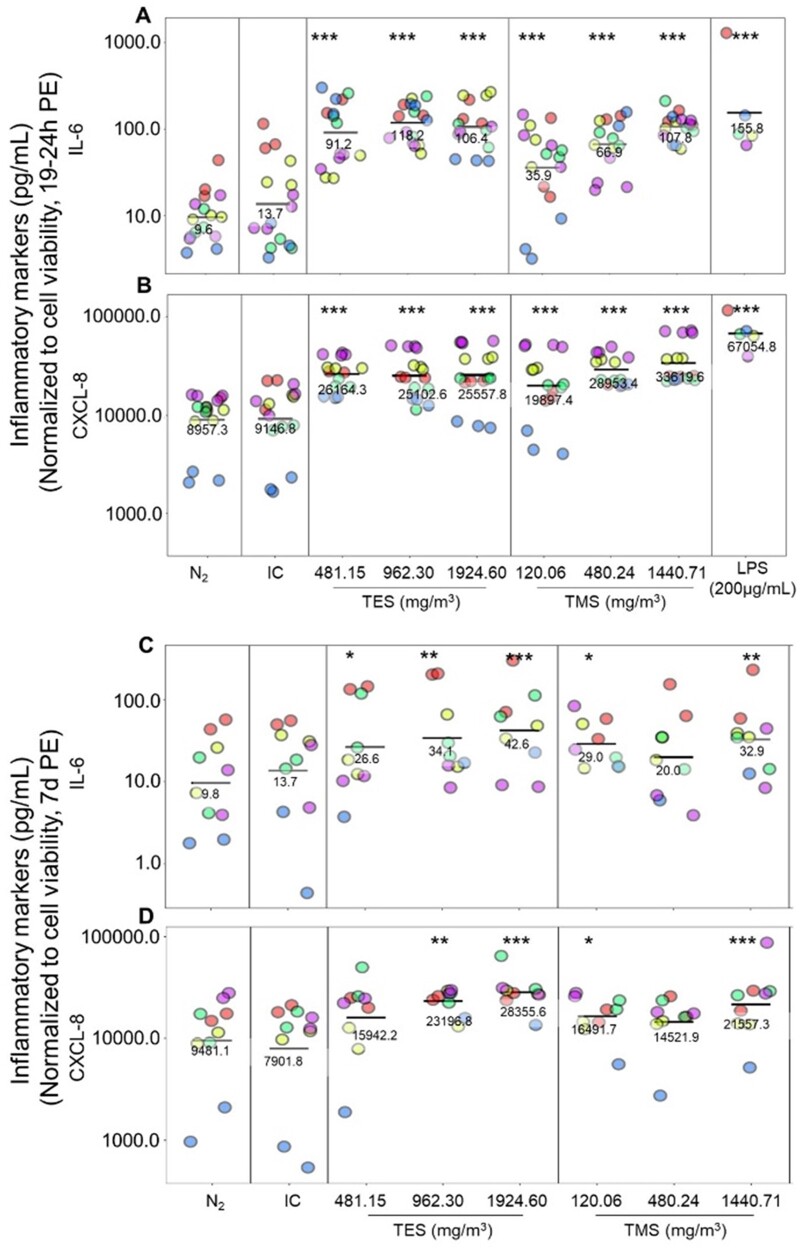
Assessment of inflammatory marker secretion in bronchial MucilAir tissues. MucilAir tissues were exposed to 481.15, 962.30, or 1924.60 mg/m^3^ TES and 120.06, 480.24, or 1440.71 mg/m^3^ TMS, for 30 min at the air-liquid interface. After 19–24 h (A for IL-6 and B for CXCL-8) or 7 days (C for IL-6 and D for CXCL-8) post-exposure, the basolateral medium was collected and stored at −80°C until analysis using Meso Scale Discovery (MSD) V-PLEX Assay. Tissues treated with lipopolysaccharide were used as a positive control for the assay. The graphs show results from 5 separate experiments in different colors. Abbreviations; IC, incubator control; LPS, lipopolysaccharide; NaCl, sodium chloride; N_2_, nitrogen gas; TES, triethoxysilane; TMS, trimethoxysilane. (*n* = 5, the asterisk [*] shows statistical significance compared with N_2_ [negative control]; *p <* *.05; **p <* *.01; ***p <* *.001).

Basolateral medium was collected at selected time points to assess the secretion of pro-inflammatory mediators. Compared with negative controls, exposure to TES or TMS increased the secretion of both cytokines, IL-6 and CXCL-8, at 19–24 h and 7 days post-exposure (Figs. 6A–D, Supplementary Figure 6). At 19–24 h post-exposure (Figs. 6A and 6B), IL-6 levels increased more than 10-fold from 9.6 pg/ml in N_2_-exposed tissues to 91.2, 118.2, and 106.4 pg/ml, respectively, for TES exposed tissues (*p* < .001). IL-6 secretion of TMS-exposed tissues increased to 35.9, 66.9, and 107.8 pg/ml, respectively. LPS-treated (200 µg/ml) tissues secreted 155.8 pg IL-6/ml. Similarly, an increase of CXCL-8 was observed but to a lesser extent from 8.96 ng/ml in N_2_-exposed tissues to 26.1, 25.1, and 25.5 ng/ml for TES-treated tissues and to 19.9, 28.9, and 33.6 ng/ml for TMS-exposed tissues. LPS treatment (200 µg/ml) increased CXCL-8 secretion to 67.1 ng/ml.

At 7 days post-exposure (Figs. 6C and 6D), the inflammatory markers are still elevated for all test conditions but in most cases to a lesser extent than at 19–24 h post exposure. IL-6 secretion was 26.6, 34.1, and 42.6 pg/ml for TES-exposed tissues and 29.0, 20.0, and 32.9 pg/ml for TMS-exposed tissues; this is about 2–4 times lower than at 19–24 h post-exposure and only about 3-fold that of N_2_-exposed tissues. CXCL-8 secretion of TES-exposed tissues was 15.9, 23.2, and 28.4 ng/ml at 7 days post-exposure and 16.5, 14.5, and 21.6 ng/ml for TMS-exposed tissues. For CXCL-8 secretion, a reduction at the low and mid concentrations of TES-exposed tissues was observed at 7 days post-exposure when compared with 19–24 h post exposure. Each concentration of TMS-exposed tissues showed lower secretion of inflammatory markers at 7 days compared with 19–24 h. Compared with the negative N_2_ control, CXCL-8 expression was still increased at 7 days post-exposure for TES and TMS. All negative controls showed similar cytokine release levels at 19–24 h and 7 days post-exposure.

#### Assessment of morphological changes in bronchial MucilAir tissues

Histology at 19–24 h and 7 days post-exposure to all treatments was conducted. Initially, the membranes with tissues were cut out of the transwell insert and shipped in an Eppendorf tube with PBS) to the location where histology was performed, which led to the membranes getting stuck to the lids of tubes. To overcome this problem, the tissues were shipped intact with the transwell insert. In addition, length of storage before conducting histology may have impacted the quality of the tissues. Due to the initial loss of several samples, only 1 full set of histology data from run 5 was obtained (depicted as purple colored dots on graphs), which was processed immediately after the experiment.

**Figure 7.**
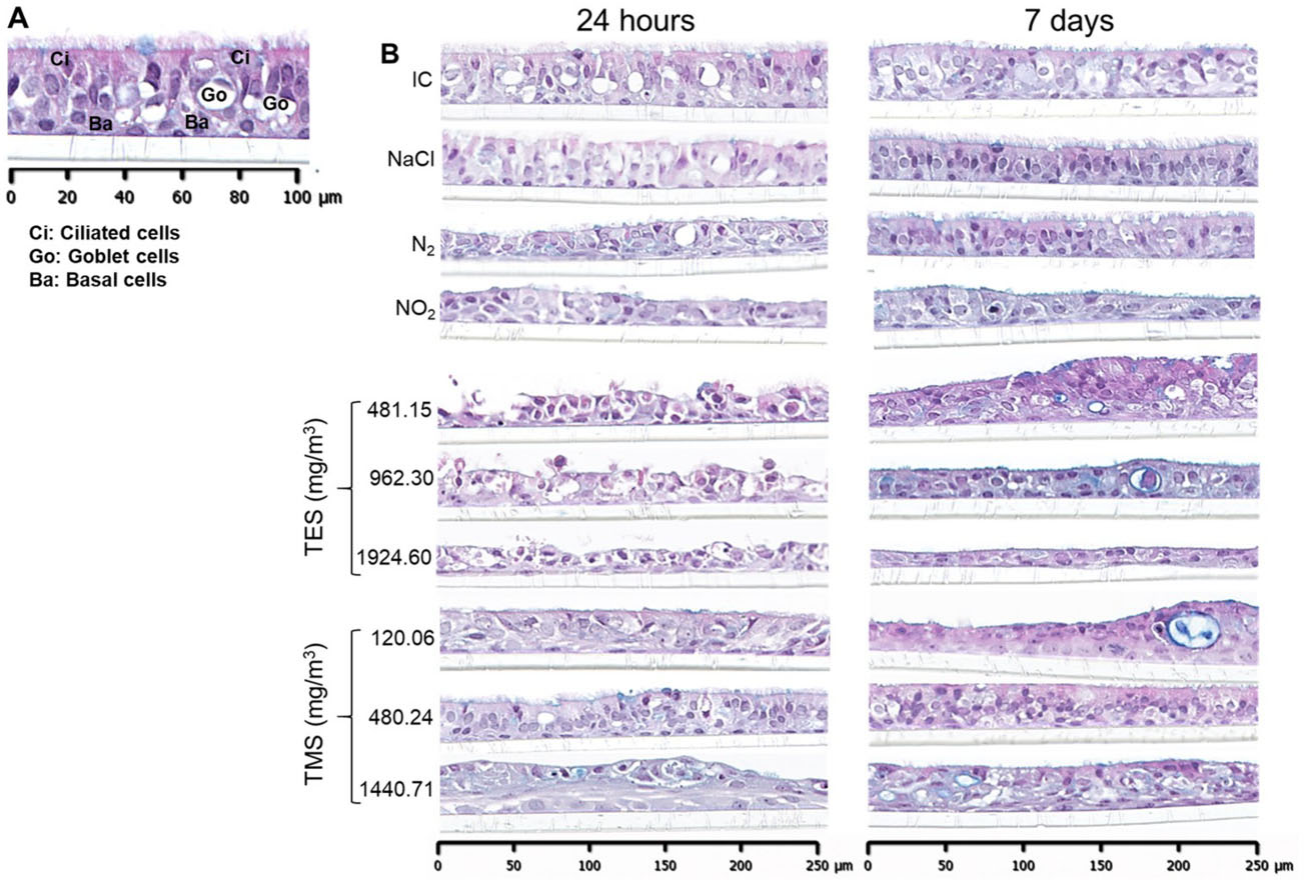
Cross-sections of bronchial MucilAir tissues. A, The cross-section of incubator control tissue with basal, goblet, and ciliated cells. B, the cross-sections MucilAir tissues exposed to controls and those exposed to 481.15, 962.30, or 1924.60 mg/m^3^ TES and 120.06, 480.24, or 1440.71 mg/m^3^ TMS for 30 min at the air-liquid interface. One donor was assessed, and 2 tissues from each time point per treatment were processed for morphological analysis. Abbreviations: IC, incubator control; N_2_, nitrogen gas; NaCl, sodium chloride; TES, triethoxysilane; TMS, trimethoxysilane.

Figure 7A shows the typical pseudostratified structure of airway epithelium comprising basal cells, ciliated cells, and mucus-secreting goblet cells. As seen in Figure 7B, no conspicuous changes were observed in the negative controls (IC, NaCl, and N2) at 24 h or 7 days. Tissues exposed to NO_2_ showed decreased cilia and structural changes. Structural damage, loss of pseudostratified structure, and decrease in cilia was observed in tissues exposed to both TES and TMS at all concentrations with TES-treated tissues showing more conspicuous effect. Particularly, exposure to TES destroyed the tissues down to the membrane whereas exposure to TMS seemed to affect the superficial layer more than the basal layer. After 7 days of exposure, none of the exposed tissues seemed to have fully recovered as compared with the negative controls.

#### Assessment of cilia beating frequency and active area in bronchial MucilAir tissues

**Figure 8.**
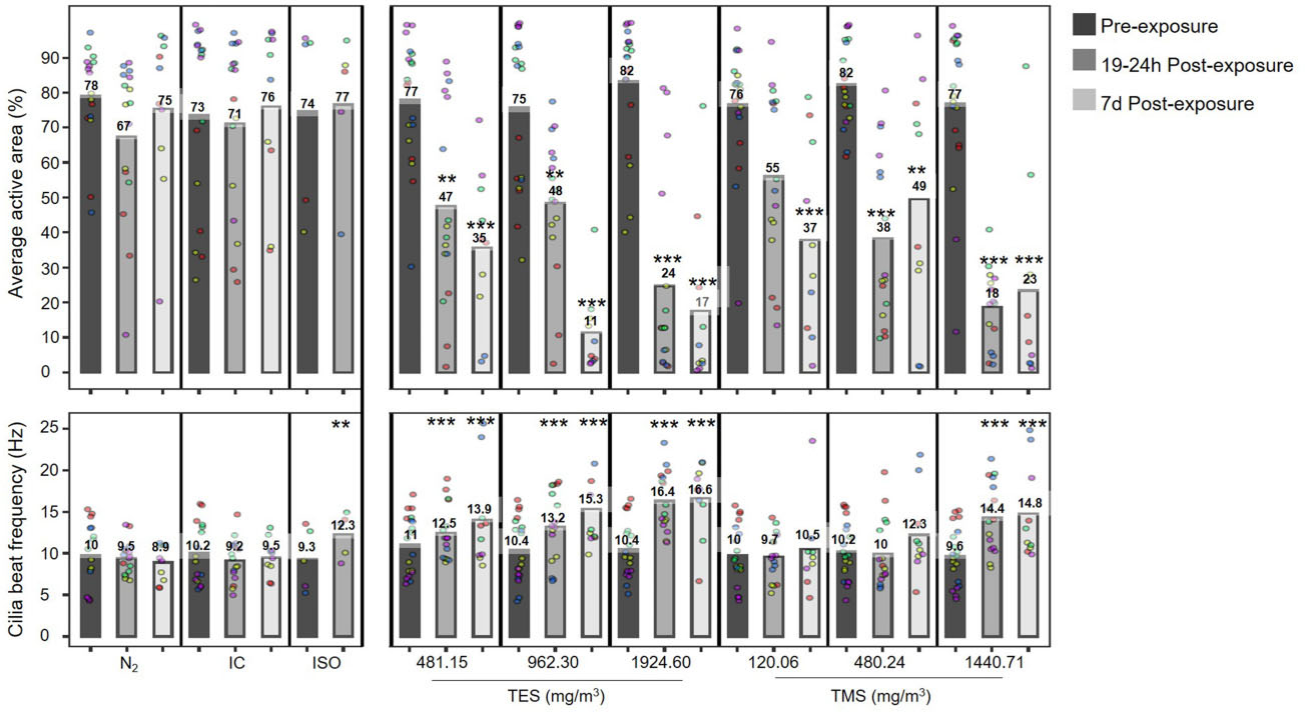
Assessment of cilia beating frequency and average active cilia beating areas in bronchial MucilAir tissues. MucilAir tissues were exposed to 481.15, 962.30, or 1924.60 mg/m^3^ TES and 120.06, 480.24, or 1440.71 mg/m^3^ TMS for 30 min at the air-liquid interface and CBF and AAA measured using Sisson-Ammons Video Analysis (SAVA) software before and 19–24 h and 7 days after exposure. The graphs show combined results from 5 separate experiments (3 different donors, 1 donor tested in 3 separate experimental runs) represented in different colors, with each run having 2 or more replicates. CBF is represented in hertz (Hz) and average AAA (%) is presented. Abbreviations: AAA, average active area; CBF, cilia beating frequency; IC, incubator control; N_2_, nitrogen gas; TES, triethoxysilane; TMS, trimethoxysilane. (*n* = 5, the asterisk [*] shows statistical significance compared with N_2_ [negative control]; *p <* *.05; **p <* *.01; ***p <* *.001).

CBF and average active area (AAA) were assessed 24 h before exposure, and 19–24 h and 7 days after exposure to TES and TMS for 30 min (Figure 8). CBF indicates how fast the cilia are beating and AAA indicates average area of the tissues with viable cilia, and together, both measurements may give an indication of the impact of exposure on muco-ciliary clearance of the tissues. Both CBF (9–10 Hz) and AAA (67%–78%) remained fairly constant throughout the experimental run for the negative controls (N_2_ and IC; Figure 8). An increase in CBF was observed in tissues treated with ISO (positive control for CBF). The data indicate that 19–24 h after exposure to silanes, the AAA was decreased compared with pre-exposure values. AAAs of tissues exposed to TES were decreased from 77%, 75%, and 82% before exposure to 47%, 48%, and 24% 19–24 h after exposure to the low, mid, and high concentration of TES, respectively. Similarly, TMS-exposed tissues showed a decreased AAA from 76%, 82%, and 77% to 55%, 38%, and 18%, respectively. Although a decrease of AAA was observed, CBF significantly increased at all concentrations of TES from 11 to 12.5 Hz, 10.4 to 13.2 Hz, and 10.4 to 16.4 Hz, respectively. However, a significant increase of CBF was only observed at the highest concentrations of TMS (from 9.6 to 14.4 Hz) at 19–24 h post-exposure.

At 7 days post-exposure, the AAA further decreased for samples exposed to low (35%), mid (11%), and high (17%) concentrations of TES, whereas the CBF increased at the same time (13.9 Hz, 15.3 Hz, and 16.6 Hz, respectively). A slight decrease in AAA at 7 days post-exposure was also observed in the low concentration TMS samples (37%) but not in the mid (49%) and high (23%) concentration samples. However, CBF did not significantly increase for low (10.5 Hz) and mid (12.3 Hz) concentrations of TMS but remained significantly elevated for the high concentration (14.8 Hz). Data per individual donor can be found in Supplementary Figure 7.

#### Assessment of barrier integrity in bronchial MucilAir tissues

**Figure 9.**
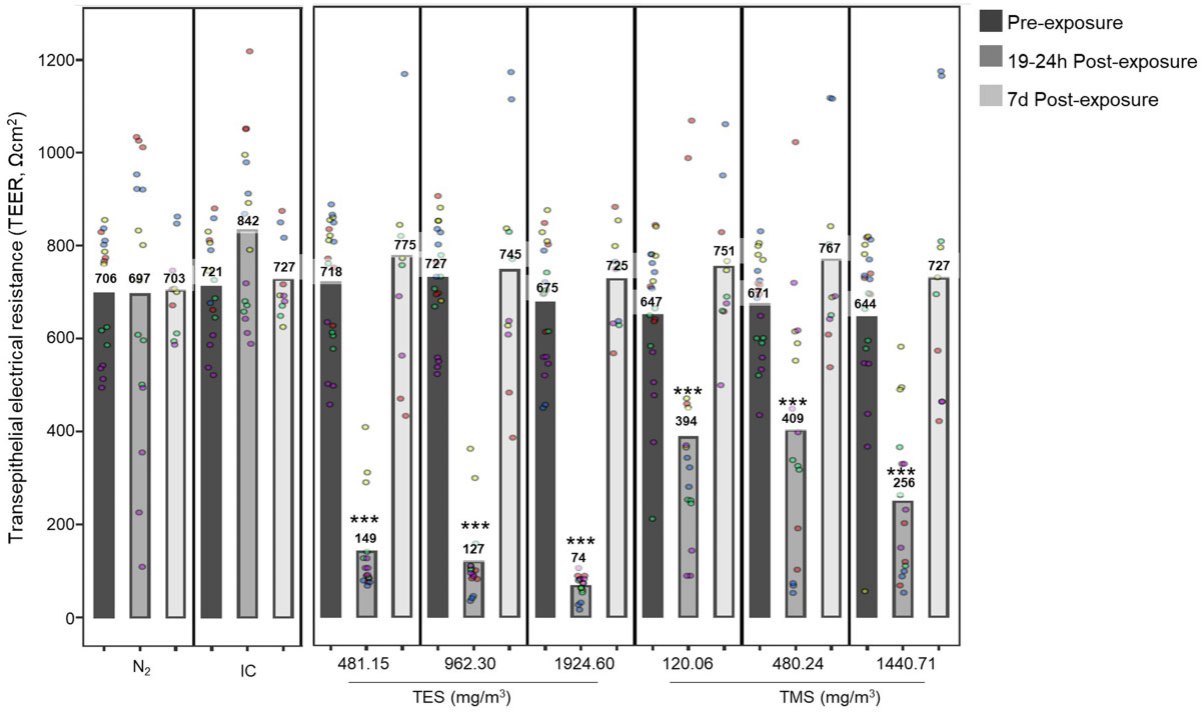
Assessment of barrier integrity in bronchial MucilAir tissues. MucilAir tissues were exposed to 481.15, 962.30, or 1924.60 mg/m^3^ TES and 120.06, 480.24, or 1440.71 mg/m^3^ TMS, for 30 min at the air-liquid interface. TEER was measured using a voltammeter before and after 19–24 h and 7 days. The graphs show combined results from 5 experimental runs (from 3 different donors) represented in different colors, with each run having at least 2 replicates. TEER is represented as Ωcm^2^. Abbreviations: IC, incubator control; N_2_, nitrogen gas; TEER, transepithelial electrical resistance; TES, triethoxysilane; TMS, trimethoxysilane. (*n* = 5, the asterisk [*] shows statistical significance compared with N_2_ [negative control]; *p <* *.05; **p <* *.01; ***p <* *.001).

Similar to all epithelial cells, *in situ* bronchial epithelial cells form a tight barrier that is maintained by the formation of tight junctions. This barrier integrity can be assessed by measuring the TEER. TEER was measured in MucilAir tissues 24 h before exposure as a quality control and 19–24 h and 7 days after exposure to TES and TMS for 30 min (Figure 9). A value >200 Ωcm^2^ is generally accepted to indicate a tight barrier for MucilAir, per the manufacturer’s recommendation. Figure 9 shows the TEER values for all runs. The TEER values for all negative controls remained similar (some minor fluctuation expected) throughout the experimental period. The electrical resistance of the epithelial barrier dropped below the threshold of 200 Ωcm^2^ for all TES-exposed tissues at 19–24 h post-exposure from 718, 727, and 675Ωcm^2^ to 149, 127, and 74 Ωcm^2^, respectively. TEER values significantly decreased for TMS-exposed tissues, but never below the threshold of 200 Ωcm^2^ from 647, 671, and 644 Ωcm^2^ to 394, 409, and 256 Ωcm^2^. Interestingly, at 7 days post-exposure, the TEER values for all tissues recovered to pre-exposure TEER values of more than 700 Ωcm^2^. Data per individual donor can be found in Supplementary Figure 8.

#### Proof-of-concept derivation of human equivalent concentration using *in vitro* data

As a proof-of-concept, the *in vitro* data generated in this study were used to derive *in vitro*-to-*in vivo* extrapolated (IVIVE) HEC. BMDS was used to derive BMCLs from the data generated in this study (McGee Hargrove *et al.*, 2021; U.S. EPA, 2022). In general, no model fit perfectly to the data because the concentration-response curve was initially set up based on 50% cell viability/cytotoxicity and not the point of departure. In BEAS-2B cells, the BMCL for TMS was lower for all cellular effects than the BMCL for TES, indicating a higher toxicity of TMS. Similarly in MucilAir, the BMCL for TMS was lower for all cellular effects except CBF and TEER (Table 5 and Supplementary Table 2A–V).

**Table 5.** Human equivalent concentrations for TES and TMS-exposed BEAS-2B and MucilAir cell systems

TES HEC Based on BMCL: BEAS-2B						TMS HEC Based on BMCL: BEAS-2B					
Cellular Effect	BMCL TES (ppm)	BMCL TES (mg/m^3^)	RGDR	30-min HEC (mg/m^3^)*^a^*	4-h HEC (mg/m^3^)	Cellular Effect	BMCL TMS (ppm)	BMCL TMS (mg/m^3^)	RGDR	30-min HEC (mg/m^3^)*^a^*	4-h HEC (mg/m^3^)
Prestoblue	6.95	44.59	0.15	6.74	0.84	Cell viability	3.91	18.78	0.15	2.84	0.35
IL-6	10.83	69.48	0.15	10.51	1.31	IL-6	9.15	43.94	0.15	6.65	0.83
CXCL-8	15.32	98.28	0.15	14.86	1.86	CXCL-8	8.53	40.96	0.15	6.19	0.77

TES HEC Based on BMCL: MucilAir						TMS HEC Based on BMCL: MucilAir					
Cellular Effect	BMCL TES (ppm)	BMCL TES (mg/m^3^)	RGDR	30-min HEC (mg/m^3^)*^a^*	4-h HEC (mg/m^3^)	Cellular Effect	BMCL TMS (ppm)	BMCL TMS (mg/m^3^)	RGDR	30-min HEC (mg/m^3^)*^a^*	4-h HEC (mg/m^3^)
Prestoblue	9.32	59.79	2.32	138.65	17.33	Cell viability	2.21	10.61	2.32	24.61	3.08
IL-6	9.65	61.91	2.32	143.55	17.94	IL-6	10.42	50.04	2.32	116.04	14.50
CXCL-8	11.28	72.36	2.32	167.80	20.98	CXCL-8	6.15	29.53	2.32	68.49	8.56
AAA	96.38	618.31	2.32	1433.76	179.22	AAA	26.99	129.62	2.32	300.56	37.57
CBF	51.91	333.02	2.32	772.22	96.53	CBF	154.8	743.40	2.32	1723.84	215.48
TEER	4.72	30.28	2.32	70.22	8.78	TEER	11.88	57.05	2.32	132.29	16.54

Abbreviations: AAA, average active area; CBF, cilia beating frequency; CXCL-8, chemokine (C-X-C motif) ligand 8; BMCL, benchmark concentration lower-confidence limit; HEC, human equivalent concentration; IL6, interleukin-6; RGDR, regional gas dose ratio; TEER, transepithelial electrical resistance; TES, triethoxysilane; TMS, trimethoxysilane.

a Final values were rounded to 2 decimal places after calculations were done.

The 4-h HEC derived from the BMCL resulted in values between 0.84 and 2.95 mg/m^3^ for TES and 0.35and 0.82 mg/m^3^ for TMS in BEAS-2B and 8.78 and 179.22 mg/m^3^ for TES and 3.08 and 215.48 mg/m^3^ for TMS in MucilAir, respectively.

## Discussion

There is a worldwide movement to develop and use reliable, human-relevant, fit-for-purpose *in vitro* testing approaches for hazard and risk assessment (Bhuller *et al.*, 2021; Craig *et al.*, 2019; Escher *et al.*, 2022; Jackson *et al.*, 2018; Stucki *et al.*, 2022; U.S. EPA, 2021; van der Zalm *et al.*, 2022). As a major route of human exposure, substantial resources have been dedicated to advancing such approaches for assessing respiratory toxicity. Using 2 silanes as test chemicals and 2 types of biological test systems, the objectives of the present study were to determine if *in vitro* test systems could evaluate the toxicity of reactive vapors, provide insights into the advantages and limitations of each test system, and to share the lessons learned that could help improve future testing.

The 2 silanes tested in this study undergo rapid hydrolysis in a humid environment and exist as vapors under atmospheric conditions, making them difficult to assess using submerged *in vitro* systems. To circumvent this issue, a dry generation set up was used to produce the silane vapor (mixed with N_2_ gas) and expose the cells at the air-liquid interface. Rapid hydrolysis also means that under *in vivo* conditions, exposure is expected to both the parent substance and the hydrolysis products—silicic acid and ethanol (for TES) or methanol (for TMS). However, because the toxicity of the parent compounds is more than 100-fold that of the hydrolysis products, silicic acid, ethanol, and methanol exposures were not included as controls in the current study (ECHA registered dossiers).

The choice of biological systems and cellular effects assessed aligned well with recent studies (Jackson *et al.*, 2018; McGee Hargrove *et al.*, 2021; Mistry *et al.*, 2020). Being portal of entry, acute irritants, the toxicity of silanes is not selective toward a specific cell type, making them amenable to testing in any biological system representing the respiratory epithelium. BEAS-2B cells and MucilAir tissues were therefore selected as test systems because of their extensive use to assess the respiratory toxicity of chemicals. The results of this study show that, for these highly reactive chemicals, cell lines such as BEAS-2B cells provide valuable information for assessing toxicity. Organotypic models such as MucilAir, offer more possibilities, such as assessing additional cellular effects (eg, TEER, CBF, AAA, and histology), and including a recovery period.

A concentration-dependent response in all cellular effects was observed when exposing BEAS-2B cells to all concentrations of either silane. For certain effects, the concentration-response was less pronounced in bronchial MucilAir tissues, indicating that BEAS-2B cells were more sensitive to silane exposure compared with MucilAir tissues. Other publications have similarly shown that BEAS-2B cells were more sensitive than MucilAir (when exposed to gasoline engine emissions; Rossner *et al.*, 2019) and EpiAirway (when exposed to methyl iodide; Mistry *et al.*, 2020). It has been suggested that these systems are more robust because, unlike monolayer cultures, the pseudostratified epithelium and the secreted mucus protect the lower cell layers from apical exposures to chemicals (Bhowmick and Gappa-Fahlenkamp, 2016; Garcia-Canton *et al.*, 2013; Han *et al.*, 2020; Rossner *et al.*, 2019).

###  

#### Interpretation of results

In BEAS-2B cells and MucilAir tissues, the results (for cytotoxicity, cell viability, and cytokine secretion), demonstrated toxicity of both tested silanes and indicated that TMS is slightly more toxic than TES. Histological analysis of MucilAir tissues showed both TES and TMS damaged the tissues as indicated by clear signs of toxicity (eg, structural changes and decrease in cilia) as compared with untreated cells. However, histology was only available from 1 experimental run. Furthermore, TES and TMS caused a concentration-dependent reduction in AAA with a corresponding increase in CBF, potentially to compensate for the loss of AAA. Because bronchial MucilAir tissues create a tight barrier, the integrity of these barriers were also assessed by measuring the TEER. Disruption of barrier integrity following TES or TMS treatment is observed at the 19–24 h time point as expected with a more substantial decrease following TES exposure for unknown reasons.

The use of MucilAir also allowed for the examination of an additional 7 day post-exposure time point. After 7 days of recovery, histological evaluation shows sustained changes to the tissue while the TEER values for all silane-exposed tissues reverted to the same or, in some cases, higher than the pre-exposure values. This is not surprising considering that the airway epithelium is a main point of contact for xenobiotics upon inhalation, therefore, the recovery of barrier function is a high priority in the initial steps of wound healing. After 7 days of recovery, AAA of TES-exposed tissues reduced even further than at 19–24 h post exposure, and CBF increased, potentially to compensate for the decreased AAA. Although an increase in CBF was observed 7 days after TMS exposure for all concentrations, only AAA for the lowest concentration decreased further than at the 19–24 h time point.

Overall, both *in vitro* systems showed that the 2 silanes are toxic, with TMS being generally more toxic than TES. As a proof of concept, this study showed a way by which *in vitro* data may be applied to calculate HECs that may inform risk decisions. The lower 4-h HEC for TMS versus TES for cell viability (PrestoBlue), cytotoxicity (LDH release), and inflammatory markers (IL-6 and CXCL-8) in BEAS-2B cells and MucilAir suggest a slightly higher toxicity of TMS than TES. This calculation takes into account the SA of the *in vitro* system and the exposure duration, which may allow better comparison of results from different studies. HECs can also be calculated from values derived using other approaches, for example, IC50 (Zhang *et al.*, 2022) or IC75 (Jackson *et al.*, 2018), previously reported in the literature.

Additional testing of other chemicals has been conducted (Jackson *et al.*, 2018; McGee Hargrove *et al.*, 2021; Mistry *et al.*, 2020) and is ongoing to better understand the applicability of *in vitro* and *in silico* systems to other chemistries. The results of this study, in conjunction with physical and chemical properties of the test substance and existing data, are a powerful tool in assessing the toxicity of inhaled chemicals. Testing known human inhalation toxicants using this approach may allow the setting of threshold values based on exposure (or human equivalent) concentrations that can then be used to categorize the toxicity of new and existing chemicals.

## Lessons learned from the study

Here, we share lessons learned throughout the study with an intent to inform future studies.

- *Concentration curves should always be established using 2 or more orthogonal assays*: Test concentrations for this study were selected based on cytotoxicity data from range-finding experiments. However, after the study began, issues were discovered related to the assay (interference with the fluorescence-based test kit and difficulty achieving 100% cytotoxicity for positive controls in MucilAir), which impacted the choice of test concentrations. This issue could have been resolved by conducting additional assay(s) for range-finding studies.
- *HEC calculations allow for comparison of results even if the tested concentrations differ between chemicals*: Although the test concentrations for TES and TMS were different in this study, HEC analysis highlighted the differences between the 2 chemicals.
- *Optimizing exposure conditions before conducting the study is essential*: The initially planned exposure duration of 1 h in preliminary experiments caused marked reduction in cell viability of BEAS-2B cells. Optimizing the exposure settings and reducing duration to 30 min prevented this decrease in cell viability.
- *Cellular concentration of the test chemical should refine concentration-response relationship:* In this study, 1 replicate per experimental run was saved for chemical analysis to determine the deposited concentration of silanes. However, due to the complexity of test chemicals being exposed as vapor and unforeseen problems during processing and preparing the tissues, the data from chemical analysis could not be used and were therefore excluded from the study. Because cellular concentration is important to dosimetry calculations and allows for more informed decisions, future studies could include such measurements.

## Conclusions

BEAS-2B cells and MucilAir can be exposed at the air-liquid interface to assess the toxicity of reactive silane vapors. The results from both cell systems demonstrate that both silanes are toxic, and TMS is more toxic than TES. Generally, the choice of which cell system to use in chemical testing will depend on the desired effects to assess the mode of action of the test chemicals, for example, whether the test chemical exerts toxicity through enzymatic (metabolic) or cellular (eg, receptor-mediated) pathways, and how the results will be used. MucilAir may offer a wider applicability domain and potentially a response that is closer to human bronchial tissue *in situ*. The ability to culture MucilAir for longer duration also allows for repeated exposure studies and the possibility to study tissue recovery. However, practical considerations, such as cost and throughput, may drive a decision to use BEAS-2B cells (or other lung cell lines). Overall, both cell-based systems proved to be a useful tool to assess inhalation toxicity.

## Supplementary Material

kfad074_Supplementary_Data

## References

[kfad074-B1] Balharry D. , Sexton K., BéruBé K. A. (2008). An in vitro approach to assess the toxicity of inhaled tobacco smoke components: Nicotine, cadmium, formaldehyde and urethane. Toxicology 244, 66–76. 18082304 10.1016/j.tox.2007.11.001

[kfad074-B2] Bates D. , Mächler M., Bolker B., Walker S. (2015). Fitting linear mixed-effects models using lme4. J. Stat. Soft. 67, 1–48. doi:10.18637/jss.v067.i01.

[kfad074-B3] Baxter A. , Thain S., Banerjee A., Haswell L., Parmar A., Phillips G., Minet E. (2015). Targeted omics analyses, and metabolic enzyme activity assays demonstrate maintenance of key mucociliary characteristics in long term cultures of reconstituted human airway epithelia. Toxicol. In Vitro 29, 864–875. 25863282 10.1016/j.tiv.2015.03.004

[kfad074-B4] Bhowmick R. , Gappa-Fahlenkamp H. (2016). Cells and culture systems used to model the small airway epithelium. Lung 194, 419–428. 27071933 10.1007/s00408-016-9875-2

[kfad074-B5] Bhuller Y. , Ramsingh D., Beal M., Kulkarni S., Gagne M., Barton-Maclaren T. S. (2021). Canadian regulatory perspective on next generation risk assessments for pest control products and industrial chemicals. Front. Toxicol. 3, 748406. 35295100 10.3389/ftox.2021.748406 PMC8915837

[kfad074-B6] Bunn A. G. (2008). A dendrochronology program library in R (dplR). Dendrochronologia 26, 115–124.

[kfad074-B7] Cesta M. C. , Zippoli M., Marsiglia C., Gavioli E. M., Mantelli F., Allegretti M., Balk R. A. (2021). The role of interleukin-8 in lung inflammation and injury: Implications for the management of COVID-19 and hyperinflammatory acute respiratory distress syndrome. Front. Pharmacol. 12, 808797. 35095519 10.3389/fphar.2021.808797 PMC8790527

[kfad074-B8] Clippinger A. J. , Allen D., Behrsing H., BéruBé K. A., Bolger M. B., Casey W., DeLorme M., Gaça M., Gehen S. C., Glover K., et al (2018a). Pathway-based predictive approaches for non-animal assessment of acute inhalation toxicity. Toxicol. In Vitro 52, 131–145. 29908304 10.1016/j.tiv.2018.06.009 PMC6760245

[kfad074-B9] Clippinger A. J. , Allen D., Jarabek A. M., Corvaro M., Gaça M., Gehen S., Hotchkiss J. A., Patlewicz G., Melbourne J., Hinderliter P., et al (2018b). Alternative approaches for acute inhalation toxicity testing to address global regulatory and non-regulatory data requirements: An international workshop report. Toxicol. In Vitro 48, 53–70. 29277654 10.1016/j.tiv.2017.12.011 PMC8215693

[kfad074-B10] Craig E. , Lowe K., Akerman G., Dawson J., May B., Reaves E., Lowit A. (2019). Reducing the need for animal testing while increasing efficiency in a pesticide regulatory setting: Lessons from the EPA Office of Pesticide Programs’ Hazard and Science Policy Council. Regul. Toxicol. Pharmacol. 108, 104481. 31546018 10.1016/j.yrtph.2019.104481 PMC7232786

[kfad074-B11] Dankers A. C. A. , Kuper C. F., Boumeester A. J., Fabriek B. O., Kooter I. M., Gröllers-Mulderij M., Tromp P., Nelissen I., Zondervan-Van Den Beuken E. K., Vandebriel R. J. (2018). A practical approach to assess inhalation toxicity of metal oxide nanoparticles in vitro. J. Appl. Toxicol. 38, 160–171. 28960351 10.1002/jat.3518

[kfad074-B12] El-Shimy W. S. , El-Dib A. S., Nagy H. M., Sabry W. (2014). A study of IL-6, IL-8, and TNF-α as inflammatory markers in COPD patients. Egypt. J. Bronchol. 8, 91–99.

[kfad074-B13] Escher S. E. , Partosch F., Konzok S., Jennings P., Luijten M., Kienhuis A., de Leeuw V. d., Reuss R., Lindemann K.-M., Bennekou S. H. (2022). Development of a roadmap for action on new approach methodologies in risk assessment. EFS3 19, 7341E.

[kfad074-B14] Foster J. R. , Cottrell R. C., Herod I. A., Atkinson H. A., Miller K. (1985). A comparative study of the pulmonary effects of NO_2_ in the rat and hamster. Br. J. Exp. Pathol. 66, 193–204. 3838681 PMC2041042

[kfad074-B15] Garcia-Canton C. , Minet E., Anadon A., Meredith C. (2013). Metabolic characterization of cell systems used in in vitro toxicology testing: Lung cell system BEAS-2B as a working example. Toxicol. In Vitro 27, 1719–1727. 23669205 10.1016/j.tiv.2013.05.001

[kfad074-B16] Goelen E. , Lambrechts M., Geyskens F., Rymen T. (1992). Development and performance characteristics of a capillary dosage unit with in situ weight sensor for the preparation of known amounts of gaseous VOC’s in air. Int. J. Environ. Anal. Chem. 47, 217–225.

[kfad074-B17] Green F. H. Y. , Vallyathan V., Hahn F. F. (2007). Comparative pathology of environmental lung disease: An overview. Toxicol. Pathol. 35, 136–147. 17325982 10.1080/01926230601132055

[kfad074-B18] Han X. , Na T., Wu T., Yuan B. Z. (2020). Human lung epithelial BEAS-2B cells exhibit characteristics of mesenchymal stem cells. PLoS One. 15, e0227174. 31900469 10.1371/journal.pone.0227174 PMC6941928

[kfad074-B19] Harkema J. R. , Carey S. A., Wagner J. G. (2006). The nose revisited: A brief review of the comparative structure, function, and toxicologic pathology of the nasal epithelium. Toxicol. Pathol. 34, 252–269. 16698724 10.1080/01926230600713475

[kfad074-B20] Hofmann W. , Asgharian B. (2003). The effect of lung structure on mucociliary clearance and particle retention in human and rat lungs. Toxicol. Sci. 73, 448–456. 12700392 10.1093/toxsci/kfg075

[kfad074-B21] Huang S. , Derouette J.-P., Constant S., Caulfuty M., Wiszniewski L. (2009). MucilAir: A novel human 3D airway epithelium model for long term toxicity testing. Toxicol. Lett. 189, S83.

[kfad074-B22] Jackson G. R. , Maione A. G., Klausner M., Hayden P. J. (2018). Prevalidation of an acute inhalation toxicity test using the EpiAirway in vitro human airway model. Appl. In Vitro Toxicol. 4, 149–158. 10.1089/aivt.2018.0004 PMC5994905 29904643

[kfad074-B23] Johnson B. I. , Cushman C. V., Lunt B. M., Kaykhaii M., Linford M. R. (2016). An introduction to silanes, their chemical vapor deposition onto Si/SiO_2_, and characterization of the resulting monolayers. Vac. Technol. Coat 17, 23–30.

[kfad074-B24] Kuznetsova A. , Brockhoff P. B., Christensen R. H. B. (2017). lmerTest package: Tests in linear mixed effects models. J. Stat. Soft. 82, 1–26.

[kfad074-B25] McGee Hargrove M. , Parr-Dobrzanski B., Li L., Constant S., Wallace J., Hinderliter P., Wolf D. C., Charlton A. (2021). Use of the MucilAir airway assay, a new approach methodology, for evaluating the safety and inhalation risk of agrochemicals. Appl. In Vitro Toxicol. 7, 50–60.

[kfad074-B26] Mistry A. , Bowen L. E., Dzierlenga M. W., Hartman J. K., Slattery S. D. (2020). Development of an in vitro approach to point-of-contact inhalation toxicity testing of volatile compounds, using organotypic culture and air-liquid interface exposure. Toxicol. In Vitro 69, 104968. 32805374 10.1016/j.tiv.2020.104968

[kfad074-B27] National Research Council (NRC) (2012a). Acute Exposure Guideline Levels for Selected Airborne Chemicals, Vol. 13. National Academies Press, Washington, DC. doi: 10.17226/15852 24830074

[kfad074-B28] National Research Council (NRC) (2012b). Acute Exposure Guideline Levels for Selected Airborne Chemicals, Vol. 11. National Academies Press, Washington, DC. doi: 10.17226/13374. 24830074

[kfad074-B29] Oesch F. , Fabian E., Landsiedel R. (2019). Xenobiotica-metabolizing enzymes in the lung of experimental animals, man and in human lung models. Arch. Toxicol. 93, 3419–3489. 31673725 10.1007/s00204-019-02602-7

[kfad074-B30] Organisation for Economic Co-operation and Development (OECD) (2009a). Test no. 403: Acute inhalation toxicity. In OECD Guidelines for the Testing of Chemicals, Section 4. OECD Publishing, Paris. doi: 10.1787/9789264070608

[kfad074-B31] Organisation for Economic Co-operation and Development (OECD) (2009b). Test no. 436: Acute inhalation toxicity – Acute toxic class method. In OECD Guidelines for the Testing of Chemicals, Section 4. OECD Publishing, Paris. doi: 10.1787/9789264076037-en

[kfad074-B32] Organisation for Economic Co-operation and Development (OECD) (2018a). Test no. 413: Subchronic inhalation toxicity: 90-day study. In OECD Guidelines for the Testing of Chemicals, Section 4. OECD Publishing, Paris. doi: 10.1787/9789264070806

[kfad074-B33] Organisation for Economic Co-operation and Development (OECD) (2018b). Test no. 433: Acute inhalation toxicity: Fixed concentration procedure. In OECD Guidelines for the Testing of Chemicals, Section 4. OECD Publishing, Paris. doi: 10.1787/9789264284166

[kfad074-B34] Organisation for Economic Co-operation and Development (OECD) (2019). Test no. 412: Subacute inhalation toxicity: 28-day study. In OECD Guidelines for the Testing of Chemicals, Section 4. OECD Publishing, Paris. doi: 10.1787/9789264070783

[kfad074-B35] Poynter M. E. , Persinger R. L., Irvin C. G., Butnor K. J., Van Hirtum H., Blay W., Heintz N. H., Robbins J., Hemenway D., Taatjes D. J., et al (2006). Nitrogen dioxide enhances allergic airway inflammation and hyperresponsiveness in the mouse. Am. J. Physiol. Lung Cell. Mol. Physiol. 290, L144–L152. 16085673 10.1152/ajplung.00131.2005

[kfad074-B36] PubChem. Triethoxysilane. Available at: https://pubchem.ncbi.nlm.nih.gov/compound/Triethoxysilane. Accessed April 18, 2023.

[kfad074-B37] Reddel R. R. , Ke Y., Gerwin B. I., McMenamin M. G., Lechner J. F., Su R. T., Brash D. E., Park J. B., Rhim J. S., Harris C. C. (1988). Transformation of human bronchial epithelial cells by infection with SV40 or adenovirus-12 SV40 hybrid virus, or transfection via strontium phosphate coprecipitation with a plasmid containing SV40 early region genes. Cancer Res. 48, 1904–1909. 2450641

[kfad074-B38] Rossner P. , Cervena T., Vojtisek-Lom M., Vrbova K., Ambroz A., Novakova Z., Elzeinova F., Margaryan H., Beranek V., Pechout M., et al (2019). The biological effects of complete gasoline engine emissions exposure in a 3D human airway model (MucilAir) and in human bronchial epithelial cells (BEAS-2B). Int. J. Mol. Sci. 20, 5710. 31739528 10.3390/ijms20225710 PMC6888625

[kfad074-B39] Sauer U. G. , Vogel S., Hess A., Kolle S. N., Ma-Hock L., van Ravenzwaay B., Landsiedel R. (2013). In vivo-in vitro comparison of acute respiratory tract toxicity using human 3D airway epithelial models and human A549 and murine 3T3 monolayer cell systems. Toxicol. In Vitro 27, 174–190. 23085368 10.1016/j.tiv.2012.10.007

[kfad074-B40] Sivars K.B. , Sivars U., Hornberg E., Zhang H., Brändén L., Bonfante R., Huang S., Constant S., Robinson I., Betts C. J., et al (2018). A 3D human airway model enables prediction of respiratory toxicity of inhaled drugs in vitro. Toxicol. Sci. 162, 301–308. 29182718 10.1093/toxsci/kfx255

[kfad074-B41] Stucki A. O. , Barton-Maclaren T. S., Bhuller Y., Henriquez J. E., Henry T. R., Hirn C., Miller-Holt J., Nagy E. G., Perron M. M., Ratzlaff D. E., et al (2022). Use of new approach methodologies (NAMs) to meet regulatory requirements for the assessment of industrial chemicals and pesticides for effects on human health. Front. Toxicol. 4, 964553. 36119357 10.3389/ftox.2022.964553 PMC9475191

[kfad074-B42] Travaglini K. J. , Nabhan A. N., Penland L., Sinha R., Gillich A., Sit R. V., Chang S., Conley S. D., Mori Y., Seita J., et al (2020). A molecular cell atlas of the human lung from single-cell RNA sequencing. Nature 587, 619–625. 33208946 10.1038/s41586-020-2922-4 PMC7704697

[kfad074-B43] United States Environmental Protection Agency (U.S. EPA) (2012). Advances in inhalation gas dosimetry for derivation of a reference concentration (RfC) and use in risk assessment. EPA/600/R-12/044. Available at: https://ordspub.epa.gov/ords/eims/eimscomm.getfile?p_download_id=514161. (accessed January 23, 2023).

[kfad074-B44] United States Environmental Protection Agency (U.S. EPA) (2021). New approach methods work plan (v2). EPA/600/X-21/209. Available at: https://www.epa.gov/system/files/documents/2021-11/nams-work-plan_11_15_21_508-tagged.pdf. (accessed January 3, 2022).

[kfad074-B45] United States Environmental Protection Agency (U.S. EPA) (2022). Benchmark dose software (BMDS) (build 3.2.0.1; model library version 2022.03). Available at: https://www.epa.gov/bmds. (accessed April 1, 2022).

[kfad074-B46] van der Zalm A. J. , Barroso J., Browne P., Casey W., Gordon J., Henry T. R., Kleinstreuer N. C., Lowit A. B., Perron M., Clippinger A. J. (2022). A framework for establishing scientific confidence in new approach methodologies. Arch. Toxicol. 96, 2865–2879. 35987941 10.1007/s00204-022-03365-4 PMC9525335

[kfad074-B47] Welch J. , Wallace J., Lansley A. B., Roper C. (2021). Evaluation of the toxicity of sodium dodecyl sulphate (SDS) in the MucilAir^TM^ human airway model in vitro. Regul. Toxicol. Pharmacol. 125, 105022. 34333067 10.1016/j.yrtph.2021.105022

[kfad074-B48] Zhang J. , Chang X., Holland T. L., Hines D. E., Karmaus A. L., Bell S., Lee K. M. (2022). Evaluation of inhalation exposures and potential health impacts of ingredient mixtures using in vitro to in vivo extrapolation. Front. Toxicol. 3, 787756. 35295123 10.3389/ftox.2021.787756 PMC8915826

