# Supplementary material for: Human cell-based in vitro systems to assess respiratory toxicity: a case study using silanes
Source: Toxicol Sci. 2023 Jul 27;195(2):213–30. doi: 10.1093/toxsci/kfad074 (PMC10535780; doi:10.1093/toxsci/kfad074)
Supplement: kfad074_Supplementary_Data [file kfad074_supplementary_data.docx]

**Human cell-based *in vitro* systems to assess respiratory toxicity: A case study using silanes**

Monita Sharma^1,*^ ^[
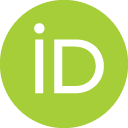
](https://orcid.org/0000-0003-2859-2581)^ , Andreas O. Stucki^1[
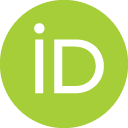
](https://orcid.org/0000-0002-0997-8502)^, Sandra Verstraelen^2[
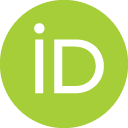
](https://orcid.org/0000-0003-0529-8208)^, Todd J. Stedeford^3[
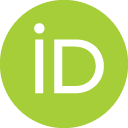
](https://orcid.org/0000-0002-3948-1919)^, An Jacobs^2^, Frederick Maes^2[
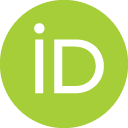
](https://orcid.org/0000-0001-7987-5931)^, David Poelmans^2^, Jo Van Laer^2^, Sylvie Remy^2[
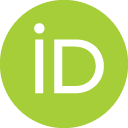
](https://orcid.org/0000-0003-0322-9620)^, Evelien Frijns^2^, David G. Allen^4[
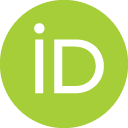
](https://orcid.org/0000-0002-8575-9547)^, Amy J. Clippinger^1[
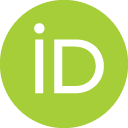
](https://orcid.org/0000-0003-3623-1196)^

^1^PETA Science Consortium International e.V., Stuttgart, Germany

^2^Sustainable HEALTH Unit, Flemish Institute for Technological Research (VITO), Mol, Belgium

^3^The Acta Group, Washington, D.C. United States

^4^Inotiv, Research Triangle Park, NC, United States

Supplementary Table 1. Information from existing *in vivo* studies conducted to assess respiratory toxicity of triethoxysilane and trimethoxysilane (Information obtained from the European Chemical Agency (ECHA) registered dossiers and OECD).

| **Triethoxysilane** | **Trimethoxysilane** |
| --- | --- |
| - **Acute toxicity** (OECD TG 403) - Rat LC50* = 500 < 1,300 mg/m^3^ (75 ppm < 195 ppm).  At 500 mg/m^3^ – During the two week post-exposure period, signs observed in animals were death, gasping, wheezing, laboured breathing, slow respiration, decreased activity, and excessive lacrimation. One male and three females were observed with red discoloration of the lung ranging from trace to moderate.  At 1,300mg/m^3^ – During the two week post-exposure period, the significant signs observed were death, decreased activity, laboured breathing, wheezing, corneal opacity, gasping, and excess lacrimation. All animals exhibited red discoloration of the lungs from mild to severe.   * ppm = mg/L x 1000 x 24.45/Mol Wt was used to convert units*.* | - **Acute toxicity** (protocol comparable to OECD TG 403) - Rat LC50 = 60 (45 to 80) ppm (ca. 300 mg/m^3^). The exposure concentrations tested were 19, 39, 71, and 166 ppm. There were deaths at all concentrations, except 19 ppm. Pathology revealed red discolouration of the lungs, dark purple discolouration of the liver, and clear fluid in the trachea and thoracic cavity. No significant difference in LC50 values between male and female rats was observed. Weight loss was observed for all exposure conditions. |
| - **Repeat dose studies**: - No repeat dose studies identified for TES. ECHA registry has data from read-across from TMS as a structural analogue or surrogate for TES (ECHA registered dossier). | - **Repeat dose studies** - Repeated (9 days) inhalation exposure of rats to TMS resulted in lethality at 5 ppm (ca. 25 mg/m^3^), with death likely due to respiratory tract injury; the no observed effect concentration (NOEC) was 0.2 ppm (ca. 1 mg/m^3^) and the low observed adverse effect concentration (LOAEC) was 1 ppm (ca. 5 mg/m^3^). Gross clinical observations indicated the respiratory system was the target organ of toxicity. - Repeated inhalation exposure of rats to TMS for 28 days at 5 and 10 ppm (ca. 25 or 50 mg/m^3^) was lethal. Exposure of rats to TMS vapor (0.02, 0.1, or 0.5 ppm; ca. 0.1, 0.5, or 2.5 mg/m^3^) for 90 days, followed by a 4-week recovery period produced no exposure-related effects. - **Repeat dose studies** - In a 90-day inhalation study (reliability score 2) conducted using a protocol comparable to OECD TG 413 and under Good Laboratory Practice (GLP), rats (10 with an additional 5 rats/sex in the control and high exposure groups) were exposed to TMS vapor for 90 days over a 13-week period for 6 hours/day, 5 days/week, followed by a 4-week recovery period. Mean exposure concentrations of 0.02, 0.10, and 0.51 ppm were achieved. This exposure regimen did not produce any exposure-related effects on clinical signs, body weight and body weight gains, food and water consumption, ophthalmic evaluations, hematology, clinical chemistry, serum protein fractions, urine chemistry, urinalysis, absolute and relative organ and tissue weights, or gross and microscopic evaluations of organs and tissues. Therefore, the NOAEC was determined to be at least 0.51 ppm under the conditions of this study. |


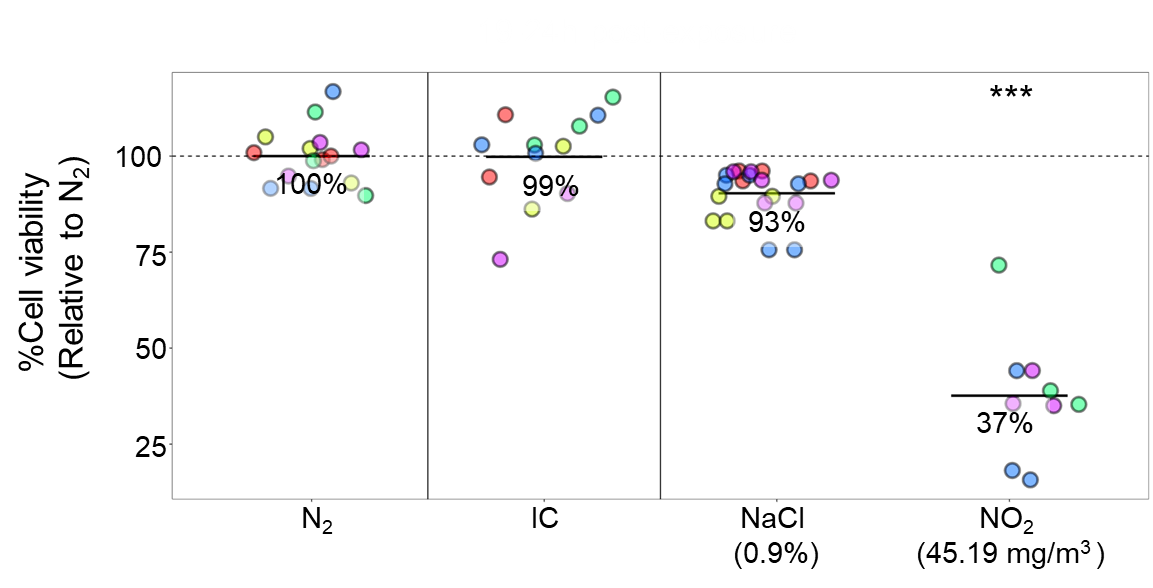


**Supplementary Figure 1. Assessment of cell viability in BEAS-2B cell line.** BEAS-2B cells were exposed to controls (N_2_, IC, NaCl, and NO_2_) for 30 minutes at the air-liquid interface and cell viability was assessed using PrestoBlue assay™ after 19-24 hours. Viability was normalized to the N_2_ control cells (= 100% cell viability). The asterisk (*) shows statistical significance compared to N_2_ (negative control); *, *P*<0.05; **, *P* < 0.01; ***, *P* < 0.001. Abbreviations: IC, incubator control; N_2_, Nitrogen gas; NaCl, sodium chloride.


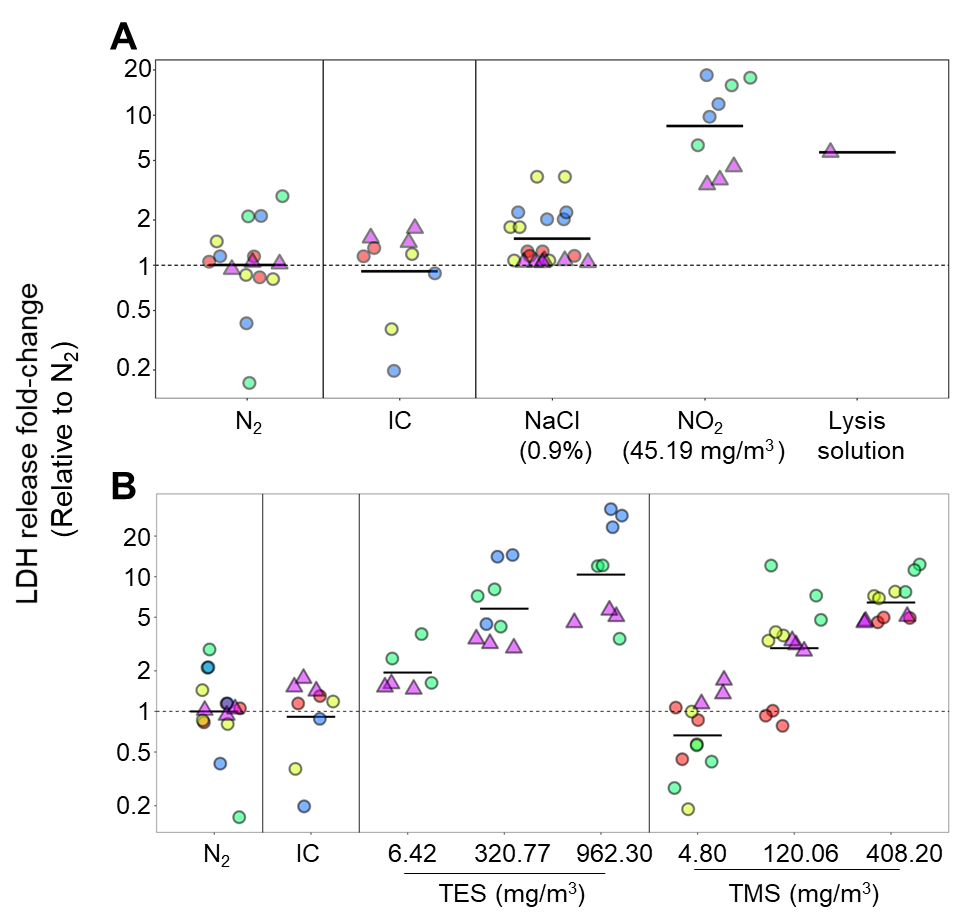


**Supplementary Figure 2. Assessment of cytotoxicity in BEAS-2B cell line.** BEAS-2B cells were exposed to controls (N_2_, IC, NaCl, NO_2,_ and lysis solution) (A) or test chemicals (B) TES (6.42, 320.77, or 962.30 mg/m^3^) or TMS (4.80, 120.06, or 408.20 mg/m^3^) vapor for 30 minutes at the air-liquid interface and cytotoxicity was assessed by measuring the LDH released from dead cells into the cell culture medium at 19-24 hours post-exposure using the absorbance-based kit (as filled triangles) and fluorescence-based kit (as filled circles). Released LDH (= cytotoxicity) is expressed as fold change in cytotoxicity relative to the N_2_ control cells. Due to issues with the fluorescence-based kit, only one data point is available for LDH positive control (lysis solution) from the absorbance-based kit. Abbreviations: IC, incubator control; N_2_, Nitrogen gas; NO_2_, nitrogen dioxide; NaCl, sodium chloride; PE, post-exposure; TES, triethoxysilane; TMS, trimethoxysilane.


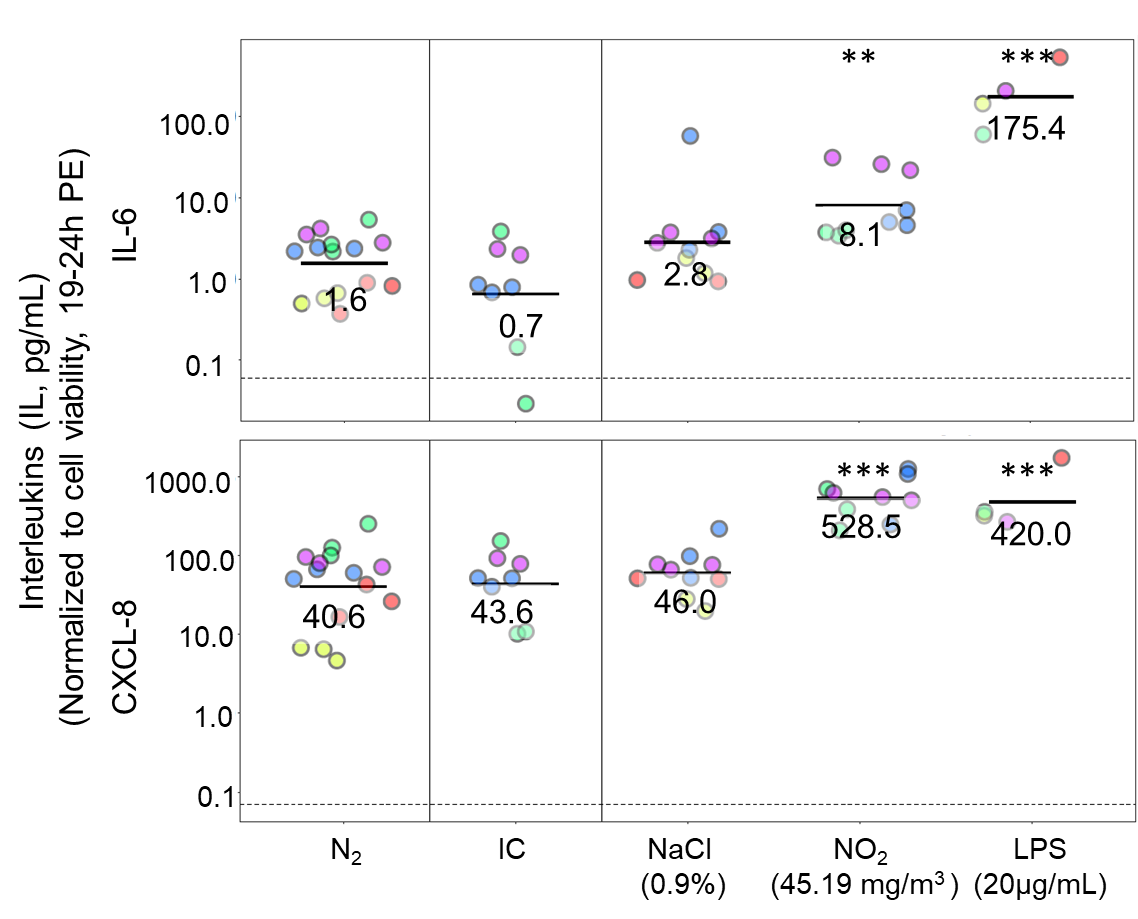


**Supplementary Figure 3. Assessment of inflammatory marker secretion in BEAS-2B cell line.** BEAS-2B cells were exposed to controls (N_2_, IC, NaCl, or NO_2_) for 30 minutes at the air-liquid interface. Samples from the basolateral medium were collected for assessment of inflammatory markers after 19-24 hours. The graphs show results from at least three separate experimental runs per condition (biological replicates; represented in different colors), with each run having 3 technical replicates. The data points show the secretion of IL-6 and CXCL-8 normalized to cell viability (PrestoBlue™ assay, Figure 3). Y-axis is shown in log-scale. The asterisk (*) shows statistical significance compared to N_2_ (negative control); *, *P*<0.05; **, *P* < 0.01; ***, *P* < 0.001. Abbreviations: IC, incubator control; IL, Interleukin (IL-6); N_2_, Nitrogen gas; NaCl, sodium chloride.


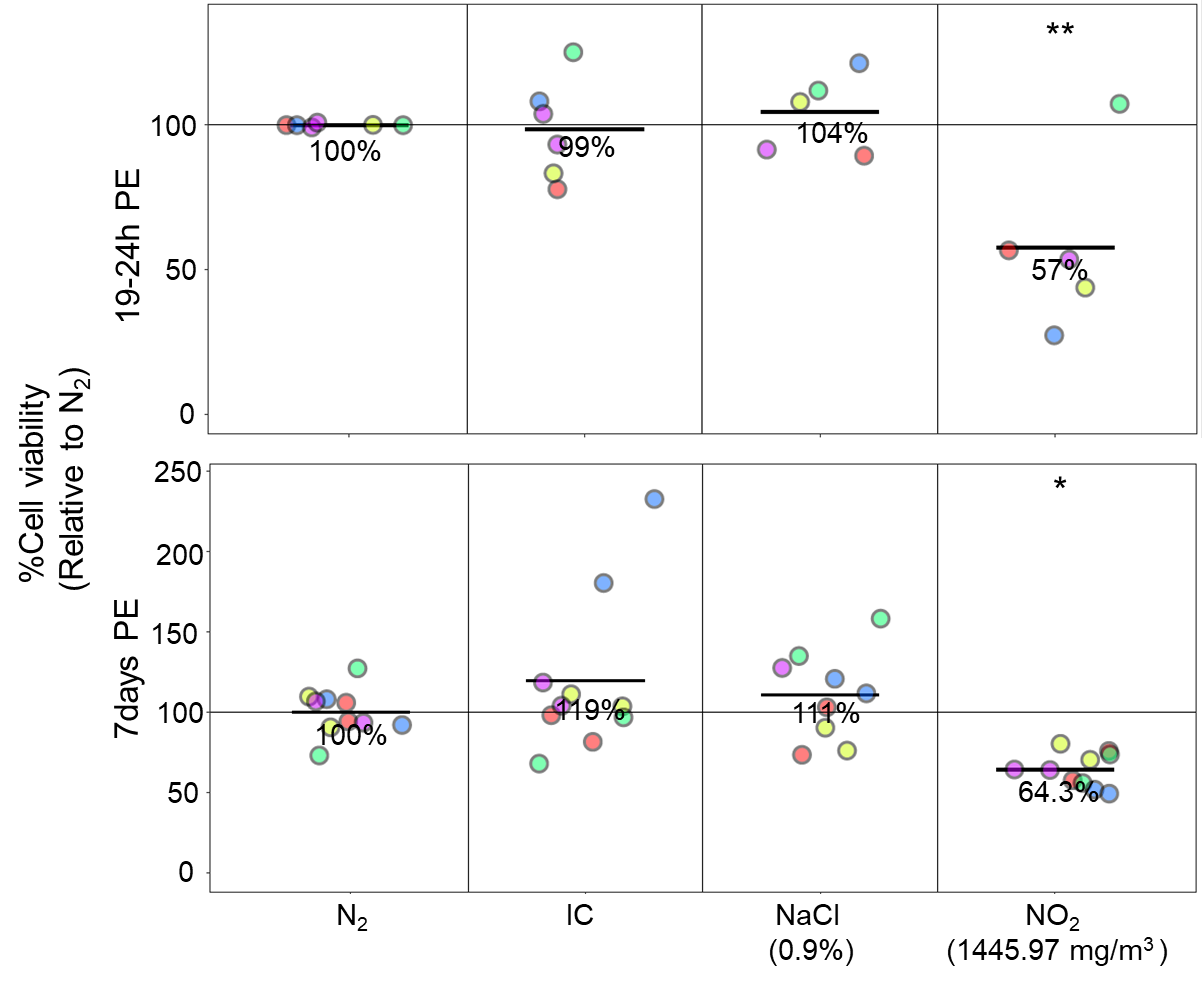


**Supplementary Figure 4. Assessment of cell viability in bronchial MucilAir™ tissues.** MucilAir™ tissues were exposed to controls (N_2_, IC, NaCl, and NO_2_) for 30 minutes at the air-liquid interface and cell viability was assessed using PrestoBlue™ assay after 19-24 hours. Viability was normalized to the N_2_ control cells (= 100% cell viability). The asterisk (*) shows statistical significance compared to N_2_ (negative control); *, *P*<0.05; **, *P* < 0.01; ***, *P* < 0.001. Abbreviations: IC, incubator control; N_2_, Nitrogen gas; NaCl, sodium chloride; NO_2_, nitrogen dioxide.


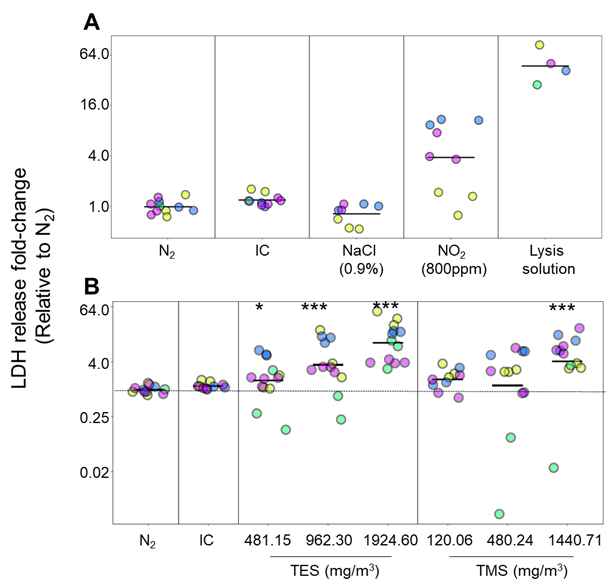


**Supplementary Figure 5. Assessment of cytotoxicity in bronchial MucilAir™ tissues.** MucilAir™ tissues were exposed to controls (A) or test chemicals (B) 481.15, 962.30, or 1924.60 mg/m^3^ TES and 120.06, 480.24, or 1440.71 mg/m^3^ TMS for 30 minutes at the air-liquid interface and cytotoxicity was assessed using lactate dehydrogenase assay after 19-24 hours. The graphs show results from separate experimental runs represented in different colors, with each run having two or more replicates. Cytotoxicity is expressed as fold change relative to the N_2_ control cells. The asterisk (*) shows statistical significance compared to N_2_ (negative control); *, *P*<0.05; **, *P* < 0.01; ***, *P* < 0.001. Abbreviations: IC, incubator control; N_2_, Nitrogen gas; NO_2_, nitrogen dioxide; NaCl, sodium chloride; PE, post-exposure; TES, triethoxysilane; TMS, trimethoxysilane.


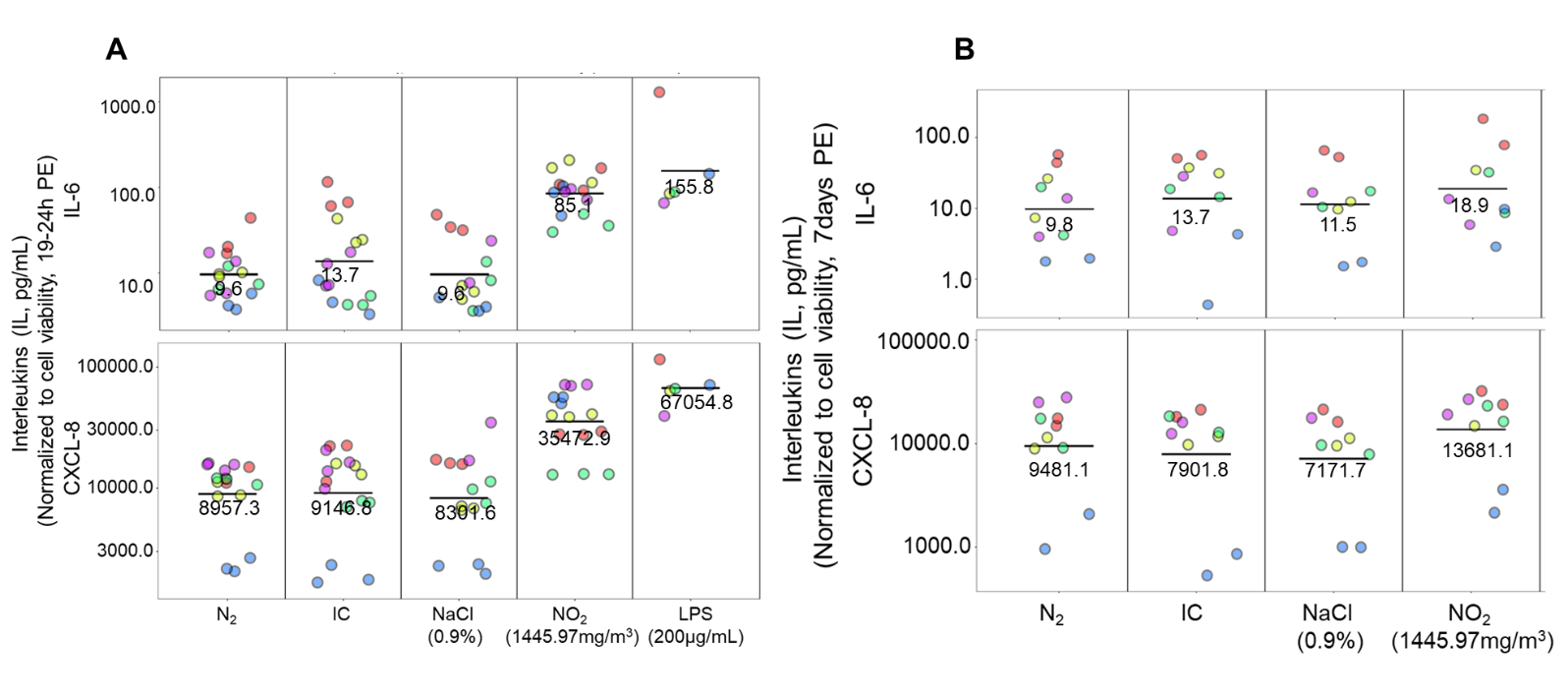


**Supplementary Figure 6. Assessment of inflammatory marker secretion in bronchial MucilAir™ tissues.** MucilAir™ tissues were exposed to (N_2_, IC, NaCl, or NO_2_) for 30 minutes at the air-liquid interface. After 19-24 hours (6A) and 7 days (6B) post-exposure, the basolateral medium was collected and stored at -80⁰C until analysis using Meso Scale Discovery (MSD) V-PLEX Assay. Tissues treated with lipo-polysaccharide were used as a positive control for the assay. The graphs show results from five separate experiments in different colors. Abbreviations; IC, incubator control; LPS, lipo-polysaccharide; NaCl, sodium chloride; N_2_, Nitrogen gas. (n=5)

**
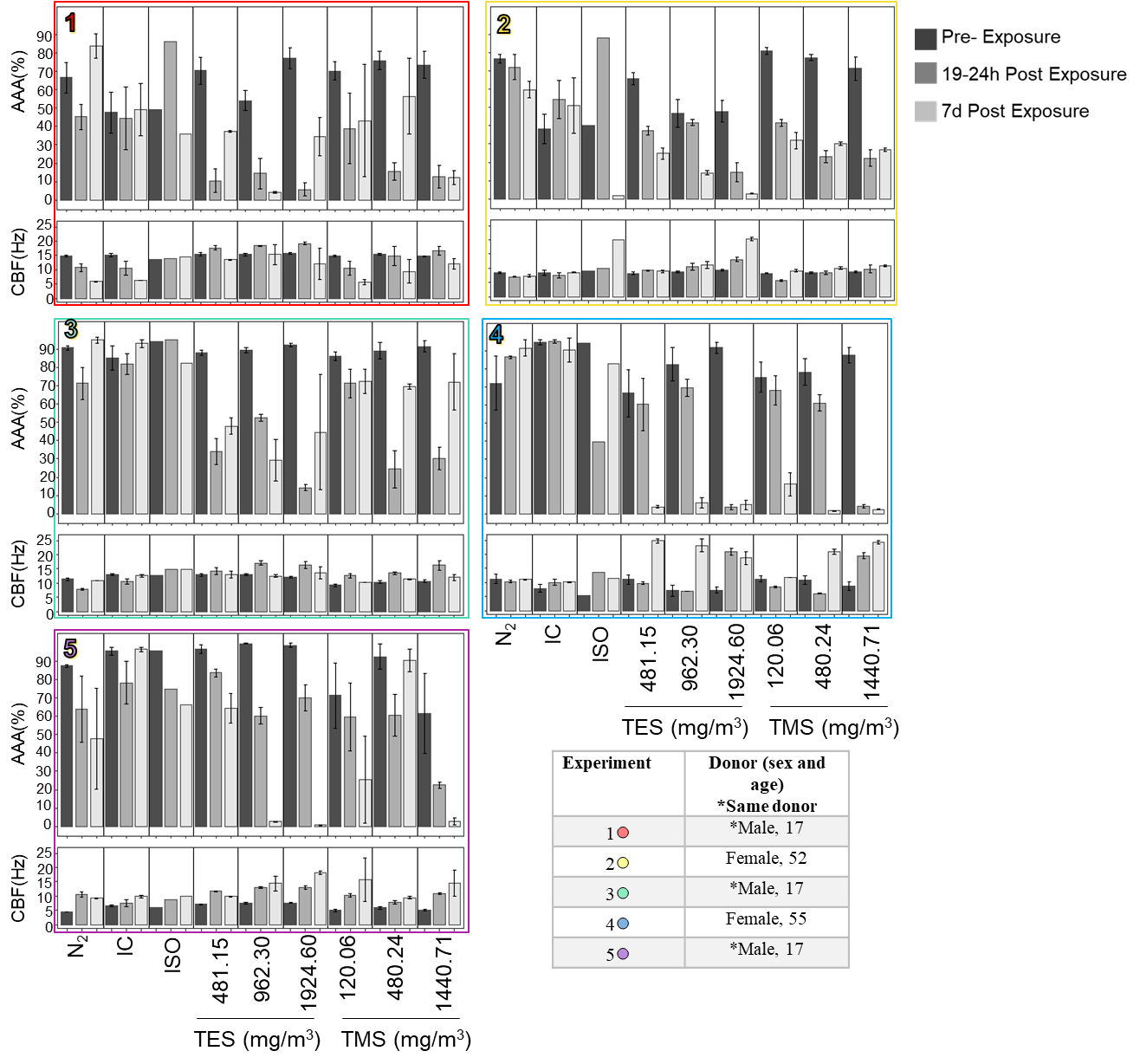
**

**Supplementary Figure 7. Assessment of cilia beating frequency and active cilia beating areas in MucilAir tissues.** MucilAir™ tissues were exposed to 481.15, 962.30, or 1924.60 mg/m^3^ TES and 120.06, 480.24, or 1440.71 mg/m^3^ TMS for 30 minutes at the air-liquid interface and CBF and AAA measured using Sisson-Ammons Video Analysis (SAVA) software before and 19-24 hours and 7 days after exposure. The graphs show results from five separate experiments (3 different donors, one donor tested three times) represented in different colors, with each run having two or more replicates. CBF is represented in hertz (Hz) and AAA (%) is presented. Abbreviations: AAA, average active area; CBF, Cilia beating frequency; IC, incubator control; N_2_, Nitrogen gas; TES, triethoxysilane; TMS, trimethoxysilane.


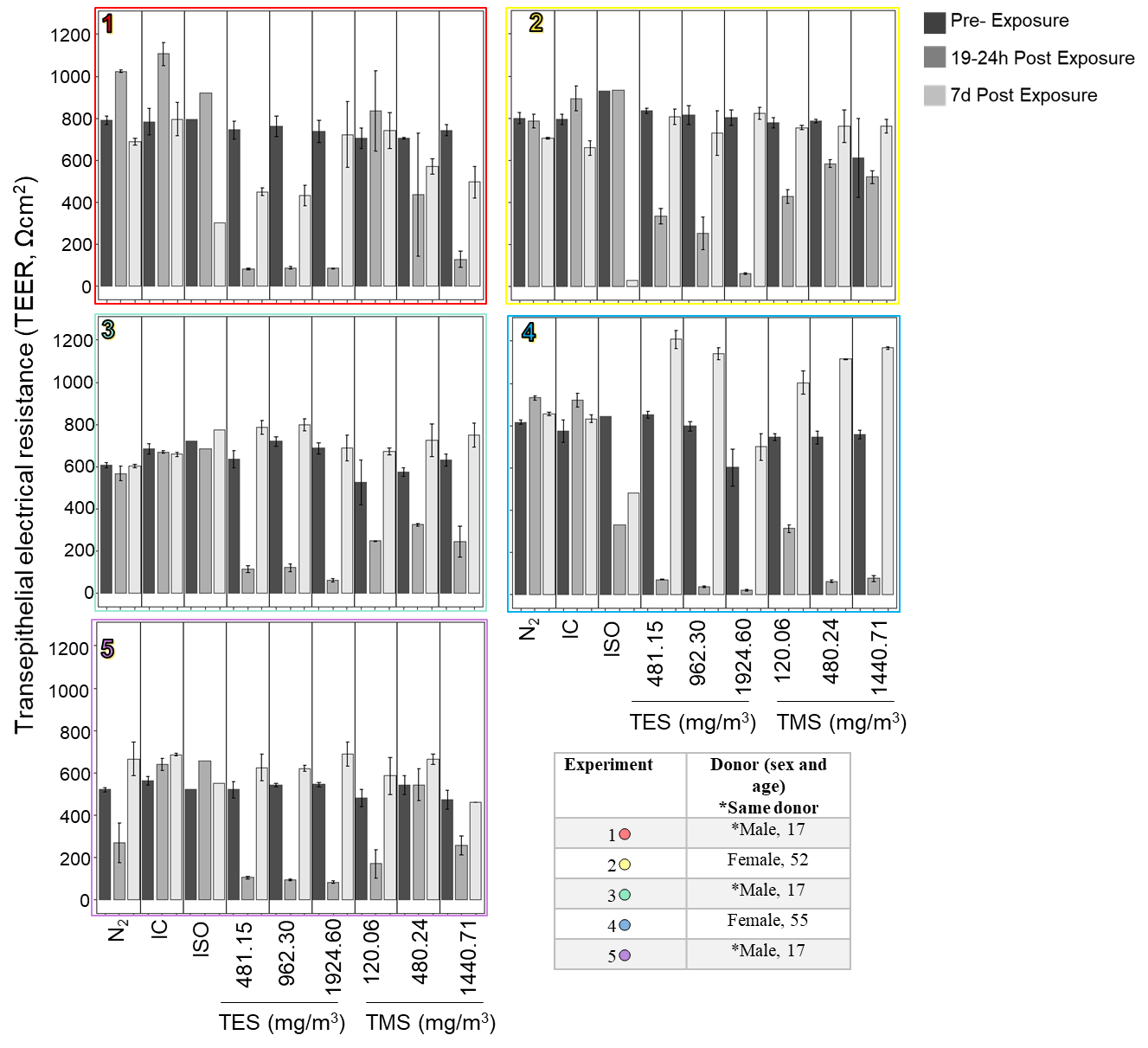


**Supplementary Figure 8. Assessment of barrier integrity in bronchial MucilAir™ tissues.** MucilAir™ tissues were exposed to 481.15, 962.30, or 1924.60 mg/m^3^ TES and 120.06, 480.24, or 1440.71 mg/m^3^ TMS for 30 minutes at the air-liquid interface. TEER was measured using a voltohmmeter before and after 19-24 hours and 7 days post exposure. The graphs show results from five separate experimental runs from three different donors, with each run having at least three replicates. TEER is represented as Ω.cm^2^. Abbreviations: IC, incubator control; N_2_, Nitrogen gas; TEER, Transepithelial electrical resistance; TES, triethoxysilane; TMS, trimethoxysilane. (n=5).

Supplementary tables 2A-2V. Tables summarizing benchmark concentration (BMC) modelling for the biological endpoints assessed in this study.

Table 2A. Summary table for BEAS-2B cells benchmark concentration modelling – Test chemical: TES; Endpoint: PrestoBlue

| Model | Scaled Residuals | | | | p-Value | AIC | BMC (ppm) | BMCL (ppm) |
| --- | --- | --- | --- | --- | --- | --- | --- | --- |
|  | At 0 ppm | At 1 ppm | At 50 ppm | At 150 ppm |  |  |  |  |
| Exponential 2 | -0.17262 | 0.71465 | -1.20168 | 1.09483 | 0.1911 | -49.962 | 12.87 | 10.06 |
| Exponential 3 | -0.17262 | 0.71464 | -1.20168 | 1.09483 | 0.1911 | -49.962 | 12.87 | 10.06 |
| **Exponential 4** | **-0.45059** | **0.60502** | **-0.03237** | **0.00905** | **0.4486** | **-50.698** | **9.65** | **6.95*** |
| Exponential 5 | -0.30160 | 0.38948 | -0.00004 | -0.00006 | NA | -49.029 | 37.02 | 7.09 |
| Hill | -0.30166 | 0.38943 | -0.00056 | -0.00070 | NA | -49.029 | 28.60 | 5.69 |
| Polynomial Degree 3 | 0.78267 | 1.21655 | -3.32830 | 1.10132 | 0.0002 | -35.664 | 28.97 | 23.71 |
| Polynomial Degree 2 | 0.78267 | 1.21655 | -3.32830 | 1.10132 | 0.0002 | -35.664 | 28.97 | 23.71 |
| Power | 0.78267 | 1.21655 | -3.32830 | 1.10132 | 0.0002 | -35.664 | 28.97 | 23.71 |
| Linear | 1.21655 | 1.21655 | -3.32830 | 1.10132 | 0.0002 | -35.664 | 28.97 | 23.71 |

*BMCL value used in Table 5

Table 2B. Summary table for BEAS-2B cells benchmark concentration modelling – Test chemical: TES; Endpoint: LDH release

| Model | Scaled Residuals | | | | p-Value | AIC | BMC (ppm) | BMCL (ppm) |
| --- | --- | --- | --- | --- | --- | --- | --- | --- |
|  | At 0 ppm | At 1 ppm | At 50 ppm | At 150 ppm |  |  |  |  |
| Exponential 2 | -0.87189 | -0.28028 | 1.14889 | -0.12198 | 0.3269 | 234.512 | 103.04 | 88.65 |
| Exponential 3 | -0.87189 | -0.28028 | 1.14889 | -0.12198 | 0.3269 | 234.512 | 103.04 | 88.65 |
| **Exponential 4** | **-0.10566** | **0.17198** | **-0.00488** | **0.00056** | **0.8399** | **234.316** | **52.85** | **24.32*** |
| Exponential 5 | -0.10548 | 0.17210 | -0.00490 | 0.00073 | 0.8399 | 234.316 | 52.85 | 24.32 |
| Hill | -0.13179 | 0.20836 | -0.00007 | 0.00023 | NA | 236.336 | 51.34 | 21.20 |
| Polynomial Degree 3 | -0.28471 | 0.07404 | 0.46126 | -0.15414 | 0.8503 | 232.599 | 66.87 | 49.59 |
| Polynomial Degree 2 | -0.28472 | 0.07403 | 0.46126 | -0.15412 | 0.8503 | 232.599 | 66.87 | 49.59 |
| Power | -0.28471 | 0.07403 | 0.46126 | -0.15413 | 0.8503 | 232.599 | 66.87 | 49.59 |
| Linear | 0.07403 | 0.07403 | 0.46125 | -0.15415 | 0.8503 | 232.599 | 66.87 | 49.59 |

*BMCL value used in Table 5

Table 2C. Summary table for BEAS-2B cells benchmark concentration modelling – Test Chemical: TES; Endpoint: IL-6

| Model | Scaled Residuals | | | p-Value | AIC | BMC (ppm) | BMCL (ppm) |  |
| --- | --- | --- | --- | --- | --- | --- | --- | --- |
|  | At 0 ppm | At 1 ppm | At 50 ppm |  |  |  |  |  |
| Exponential 2 | 0.02572 | -0.03153 | 0.00009 | 0.96754 | 307.5860 | 33.85 | 26.97 |  |
| Exponential 3 | 0.02576 | -0.03150 | -0.00002 | NA | 309.5860 | 33.85 | 26.97 |  |
| Exponential 4 | 0.11891 | -0.17158 | 0.00741 | NA | 309.6281 | 13.15 | 1.75 |  |
| Exponential 5 | 0.01586 | -0.02292 | -0.00002 | NA | 311.5851 | 26.56 | 1.39 |  |
| Hill | 0.01571 | -0.01962 | -0.00063 | NA | 311.5850 | 8.00 | 1.05 |  |
| Polynomial Degree 2 | 0.11961 | -0.15654 | -0.00033 | NA | 309.6232 | 13.80 | 10.83 |  |
| Power | 0.01739 | -0.01832 | 0.00009 | NA | 309.5850 | 46.82 | 44.70 |  |
| **Linear** | **-0.15711** | **-0.15711** | **0.00342** | **0.84375** | **307.6232** | **13.80** | **10.83** |  |

*BMCL value used in Table 5

Table 2D. Summary table for BEAS-2B cells benchmark concentration modelling – Test Chemical: TES; Endpoint: CXCL-8 (IL-8)

| Model | Scaled Residuals | | | p-Value | AIC | BMC (ppm) | BMCL (ppm) |  |
| --- | --- | --- | --- | --- | --- | --- | --- | --- |
|  | At 0 ppm | At 1 ppm | At 50 ppm |  |  |  |  |  |
| Exponential 2 | 0.04002 | -0.04924 | 0.00004 | 0.94941 | 498.0247 | 35.79 | 30.40 |  |
| Exponential 3 | 0.04003 | -0.04924 | -0.00025 | NA | 500.0247 | 35.79 | 30.40 |  |
| Exponential 4 | 0.10405 | -0.12041 | 0.00242 | NA | 500.0460 | 20.41 | 15.49 |  |
| Exponential 5 | 0.02632 | -0.03350 | 0.00012 | NA | 500.0224 | 7.23 | 1.52 |  |
| Hill | 0.02612 | -0.03365 | -0.00006 | NA | 502.0224 | 7.61 | 1.05 |  |
| Polynomial Degree 2 | 0.09680 | -0.12545 | 0.00162 | NA | 500.0458 | 20.46 | 15.32 |  |
| Power | 0.02620 | -0.03359 | -0.00038 | NA | 500.0224 | 48.15 | 47.19 |  |
| **Linear** | **-0.12623** | **-0.12623** | **0.00252** | **0.87404** | **498.0458** | **20.46** | **15.32*** |  |

*BMCL value used in Table 5

Table 2E. Summary table for BEAS-2B cells benchmark concentration modelling – Test chemical: TMS; Endpoint: LDH release

| Model | Scaled Residuals | | | | p-Value | AIC | BMC  (ppm) | BMCL  (ppm) |
| --- | --- | --- | --- | --- | --- | --- | --- | --- |
|  | At 0 ppm | At 1 ppm | At 25 ppm | At 85 ppm |  |  |  |  |
| Exponential 2 | -1.13928 | -1.82921 | 2.87563 | -0.29788 | 0.0006 | 167.345 | 36.73 | 32.24 |
| Exponential 3 | -1.13926 | -1.82917 | 2.87547 | -0.29800 | 0.0006 | 167.345 | 36.73 | 32.24 |
| **Exponential 4** | **0.58029** | **-0.69224** | **0.05153** | **-0.00808** | **0.3636** | **155.163** | **10.45** | **7.12*** |
| Exponential 5 | 0.42545 | -0.47549 | -0.00065 | 0.00019 | NA | 156.746 | 19.90 | 7.33 |
| Hill | 0.42713 | -0.47574 | 0.00025 | -0.00045 | NA | 156.748 | 16.87 | 6.79 |
| Polynomial Degree 3 | -0.17701 | -1.15383 | 1.89572 | -0.54399 | 0.0626 | 157.881 | 19.08 | 15.77 |
| Polynomial Degree 2 | -0.17701 | -1.15383 | 1.89572 | -0.54399 | 0.0626 | 157.881 | 19.08 | 15.77 |
| Power | -0.17701 | -1.15383 | 1.89572 | -0.54399 | 0.0626 | 157.881 | 19.08 | 15.77 |
| Linear | -1.15383 | -1.15383 | 1.89572 | -0.54399 | 0.0626 | 157.881 | 19.08 | 15.77 |

*BMCL value used in Table 5

Table 2F. Summary table for BEAS-2B cells benchmark concentration modelling – Test chemical: TMS; Endpoint: PrestoBlue

| Model | Scaled Residuals | | | | p-Value | AIC | BMC  (ppm) | BMCL  (ppm) |
| --- | --- | --- | --- | --- | --- | --- | --- | --- |
|  | At 0 ppm | At 1 ppm | At 25 ppm | At 85 ppm |  |  |  |  |
| Exponential 2 | -0.83862 | 2.24239 | -2.51422 | 1.63744 | 0.0002 | -51.536 | 10.17 | 8.01 |
| Exponential 3 | -0.83862 | 2.24238 | -2.51421 | 1.63744 | 0.0002 | -51.536 | 10.17 | 8.01 |
| **Exponential 4** | **-1.70275** | **2.06280** | **-0.21853** | **0.05935** | **0.0053** | **-59.157** | **5.37** | **3.91*** |
| Exponential 5 | -1.47344 | 1.64734 | 0.00006 | -0.00006 | NA | -59.790 | 21.11 | 4.99 |
| Hill | -1.47340 | 1.64731 | -0.00073 | -0.00017 | NA | -59.790 | 16.70 | 4.59 |
| Polynomial Degree 3 | 0.13102 | 2.60622 | -3.85623 | 1.10352 | <0.0001 | -38.529 | 19.87 | 16.38 |
| Polynomial Degree 2 | 0.13102 | 2.60622 | -3.85623 | 1.10352 | <0.0001 | -38.529 | 19.87 | 16.38 |
| Power | 0.13102 | 2.60622 | -3.85623 | 1.10352 | <0.0001 | -38.529 | 19.87 | 16.37 |
| Linear | 2.60622 | 2.60622 | -3.85623 | 1.10352 | <0.0001 | -38.529 | 19.87 | 16.38 |

*BMCL value used in Table 5

Table 2G. Summary table for BEAS-2B cells benchmark concentration modelling – Test Chemical: TMS; Endpoint: IL-6

| Model | Scaled Residuals | | | p-Value | AIC | BMC  (ppm) | BMCL  (ppm) |
| --- | --- | --- | --- | --- | --- | --- | --- |
|  | At 0 ppm | At 1 ppm | At 25 ppm |  |  |  |  |
| Exponential 2 | 0.18625 | -0.21976 | 0.00138 | 0.77317 | 301.6213 | 19.04 | 16.56 |
| Exponential 3 | 0.21176 | -0.19154 | -0.00285 | NA | 303.6198 | 19.19 | 16.56 |
| Exponential 4 | 0.29466 | -0.36070 | 0.01571 | NA | 303.7560 | 11.91 | 2.16 |
| Exponential 5 | 0.16330 | -0.18250 | -0.00153 | NA | 305.5983 | 19.76 | 1.21 |
| Hill | 0.16312 | -0.18237 | 0.00000 | NA | 305.5982 | 5.11 | 1.07 |
| **Polynomial Degree 2** | **0.16854** | **-0.18874** | **0.00030** | **0.80016** | **301.6023** | **17.56** | **9.15*** |
| Power | 0.16312 | -0.18237 | 0.00000 | NA | 303.5982 | 24.09 | 9.15 |
| Linear | -0.34614 | -0.34614 | 0.01369 | 0.64755 | 301.7472 | 12.18 | 9.08 |

*BMCL value used in Table 5

Table 2H. Summary table for BEAS-2B cells benchmark concentration modelling – Test Chemical: TMS; Endpoint: CXCL-8 (IL-8)

| Model | Scaled Residuals | | | p-Value | AIC | BMC  (ppm) | BMCL  (ppm) |
| --- | --- | --- | --- | --- | --- | --- | --- |
|  | At 0 ppm | At 1 ppm | At 25 ppm |  |  |  |  |
| Exponential 2 | 0.66624 | -0.72367 | 0.00543 | 0.32225 | 478.0199 | 16.12 | 14.01 |
| Exponential 3 | 0.59667 | -0.66820 | 0.00003 | NA | 479.8509 | 21.80 | 14.07 |
| Exponential 4 | 0.73325 | -0.85404 | 0.03430 | NA | 480.3294 | 10.99 | 8.35 |
| Exponential 5 | 0.59726 | -0.66759 | 0.00011 | NA | 481.8508 | 6.64 | 1.23 |
| Hill | 0.59717 | -0.66765 | -0.00001 | NA | 481.8508 | 5.44 | 1.09 |
| **Polynomial Degree 2** | **0.60273** | **-0.67495** | **0.00108** | **0.36298** | **477.8676** | **16.63** | **8.53*** |
| Power | 0.59717 | -0.66765 | 0.00000 | NA | 479.8508 | 24.20 | 8.54 |
| Linear | -0.85366 | -0.85366 | 0.03415 | 0.25637 | 478.3283 | 11.00 | 8.35 |

*BMCL value used in Table 5

Table 2I. Summary table for MucilAir benchmark concentration modelling – Test chemical: TES; Endpoint: LDH release

| Model | Scaled Residuals | | | | p-Value | AIC | BMC (ppm) | BMCL (ppm) |
| --- | --- | --- | --- | --- | --- | --- | --- | --- |
|  | At 0 ppm | At 75 ppm | At 150 ppm | At 300 ppm |  |  |  |  |
| Exponential 2 | -1.06702 | 0.02032 | 0.89981 | -0.19581 | 0.3642 | 445.816 | 233.53 | 204.76 |
| Exponential 3 | -1.06714 | 0.02020 | 0.89970 | -0.19576 | 0.3642 | 445.816 | 233.53 | 204.76 |
| **Exponential 4** | **0.03672** | **-0.09453** | **0.07917** | **-0.01492** | **0.8969** | **445.813** | **147.21** | **85.86*** |
| Exponential 5 | 0.03823 | -0.07386 | 0.07870 | -0.02009 | NA | 447.810 | 147.13 | 85.96 |
| Hill | -0.00011 | 0.00039 | -0.00054 | -0.00083 | NA | 447.796 | 145.76 | 73.60 |
| Polynomial Degree 3 | -0.11995 | -0.00903 | 0.24581 | -0.11676 | 0.9567 | 443.885 | 160.95 | 120.07 |
| Polynomial Degree 2 | -0.11993 | -0.00903 | 0.24581 | -0.11678 | 0.9567 | 443.885 | 160.95 | 120.07 |
| Power | -0.11996 | -0.00904 | 0.24581 | -0.11674 | 0.9567 | 443.885 | 160.95 | 121.16 |
| Linear | -0.00903 | -0.00903 | 0.24581 | -0.11676 | 0.9567 | 443.885 | 160.95 | 120.07 |

*BMCL value used in Table 5

Table 2J. Summary table for MucilAir benchmark concentration modelling – Test chemical: TES; Endpoint: PrestoBlue

| Model | Scaled Residuals | | | | p-Value | AIC | BMC (ppm) | BMCL (ppm) |
| --- | --- | --- | --- | --- | --- | --- | --- | --- |
|  | At 0 ppm | At 75 ppm | At 150 ppm | At 300 ppm |  |  |  |  |
| Exponential 2 | 0.90485 | -1.22598 | -0.18928 | 0.53628 | 0.2463 | 201.864 | 117.43 | 73.28 |
| Exponential 3 | 0.90486 | -1.22598 | -0.18928 | 0.53627 | 0.2463 | 201.864 | 117.43 | 73.28 |
| **Exponential 4** | **0.09359** | **-0.46134** | **0.61262** | **-0.24487** | **0.4144** | **201.727** | **48.17** | **9.32*** |
| Exponential 5 | 0.09359 | -0.46134 | 0.61262 | -0.24487 | 0.4144 | 201.727 | 48.17 | 9.32 |
| Hill | 0.03496 | -0.32876 | 0.53082 | -0.23706 | 0.5016 | 201.513 | 40.42 | 0.00 |
| Polynomial Degree 3 | 1.12889 | -1.25982 | -0.36804 | 0.49897 | 0.1749 | 202.549 | 141.36 | 94.78 |
| Polynomial Degree 2 | 1.12889 | -1.25982 | -0.36804 | 0.49897 | 0.1749 | 202.549 | 141.36 | 94.78 |
| Power | 1.12889 | -1.25981 | -0.36804 | 0.49897 | 0.1749 | 202.549 | 141.36 | 94.78 |
| Linear | -1.25981 | -1.25981 | -0.36804 | 0.49897 | 0.1749 | 202.549 | 141.36 | 94.78 |

*BMCL value used in Table 5

Table 2K. Summary table for MucilAir benchmark concentration modelling – Test Chemical: TES; Endpoint: IL-6

| Model | Scaled Residuals | | | | p-Value | AIC | BMC (ppm) | BMCL (ppm) |
| --- | --- | --- | --- | --- | --- | --- | --- | --- |
|  | At 0 ppm | At 75 ppm | At 150 ppm | At 300 ppm |  |  |  |  |
| Exponential 2 | -3.02059 | 2.06424 | 1.70680 | -0.88688 | <0.0001 | 731.3792 | 308.95 | 233.84 |
| Exponential 3 | -3.01937 | 2.06479 | 1.70719 | -0.88704 | <0.0001 | 731.3792 | 308.90 | 233.84 |
| **Exponential 4** | **0.00077** | **-0.02943** | **0.21422** | **-0.18651** | **0.77514** | **713.5380** | **18.73** | **9.65*** |
| Exponential 5 | 0.00029 | -0.01993 | 0.20993 | -0.19125 | NA | 715.5374 | 19.37 | 9.69 |
| Hill | 0.00000 | 0.00191 | 0.19535 | -0.19512 | NA | 715.5326 | 57.61 | 0.00 |
| Polynomial Degree 2 | -2.41004 | 2.26463 | 1.53099 | -1.31367 | 0.00019 | 728.5967 | 239.64 | 163.13 |
| Power | -2.41004 | 2.26463 | 1.53099 | -1.31367 | 0.00019 | 728.5967 | 239.64 | 163.13 |
| Linear | 2.26463 | 2.26463 | 1.53099 | -1.31367 | 0.00019 | 728.5967 | 239.63 | 163.13 |

*BMCL value used in Table 5

Table 2L. Summary table for MucilAir benchmark concentration modelling – Test Chemical: TES; Endpoint: CXCL-8 (IL-8)

| Model | Scaled Residuals | | | | p-Value | AIC | BMC (ppm) | BMCL (ppm) |
| --- | --- | --- | --- | --- | --- | --- | --- | --- |
|  | At 0 ppm | At 75 ppm | At 150 ppm | At 300 ppm |  |  |  |  |
| Exponential 2 | -2.34277 | 1.92255 | 1.07939 | -0.70083 | 0.00261 | 1380.8458 | 276.21 | 209.22 |
| Exponential 3 | -2.34283 | 1.92252 | 1.07938 | -0.70080 | 0.00261 | 1380.8458 | 276.21 | 209.22 |
| Exponential 4 | -0.00573 | 0.10284 | -0.42805 | 0.33431 | 0.57994 | 1371.2552 | 34.89 | 11.28 |
| **Exponential 5** | **-0.00567** | **0.10161** | **-0.42774** | **0.33506** | **0.57994** | **1371.2552** | **34.86** | **11.28*** |
| Hill | -0.00175 | 0.13406 | -0.31913 | 0.19107 | 0.69238 | 1371.1053 | 24.33 | 0.00 |
| Polynomial Degree 2 | -1.90466 | 2.01043 | 0.88950 | -0.93146 | 0.00642 | 1379.0447 | 230.16 | 158.49 |
| Power | -1.90465 | 2.01045 | 0.88953 | -0.93142 | 0.00642 | 1379.0447 | 230.16 | 158.49 |
| Linear | 2.01045 | 2.01045 | 0.88953 | -0.93142 | 0.00642 | 1379.0447 | 230.16 | 158.49 |

*BMCL value used in Table 5

Table 2M. Summary table for MucilAir benchmark concentration modelling – Test Chemical: TES; Endpoint: AAA

| Model | Scaled Residuals | | | | p-Value | AIC | BMC (ppm) | BMCL (ppm) |
| --- | --- | --- | --- | --- | --- | --- | --- | --- |
|  | At 0 ppm | At 75 ppm | At 150 ppm | At 300 ppm |  |  |  |  |
| **Exponential 2** | **0.19650** | **-0.85423** | **0.98820** | **-0.35538** | **0.3869** | **599.184** | **157.59** | **96.38*** |
| Exponential 3 | 0.19650 | -0.85423 | 0.98820 | -0.35538 | 0.3869 | 599.184 | 157.59 | 96.38 |
| Exponential 4 | 0.19650 | -0.85423 | 0.98820 | -0.35538 | 0.3869 | 599.184 | 157.59 | 96.38 |
| Exponential 5 | 0.19650 | -0.85423 | 0.98820 | -0.35538 | 0.3869 | 599.184 | 157.59 | 96.38 |
| Hill | 0.32044 | -0.97522 | 0.82768 | -0.17282 | 0.1805 | 601.079 | 171.12 | 68.87 |
| Polynomial Degree 3 | 0.51632 | -1.09731 | 0.61331 | -0.03233 | 0.3916 | 599.160 | 190.27 | 137.68 |
| Polynomial Degree 2 | 0.51632 | -1.09731 | 0.61331 | -0.03233 | 0.3916 | 599.160 | 190.27 | 137.68 |
| Power | 0.51632 | -1.09731 | 0.61331 | -0.03233 | 0.3916 | 599.160 | 190.27 | 137.68 |
| Linear | -1.09731 | -1.09731 | 0.61331 | -0.03233 | 0.3916 | 599.160 | 190.27 | 137.68 |

*BMCL value used in Table 5

Table 2N. Summary table for MucilAir benchmark concentration modelling – Test Chemical: TES; Endpoint: CBF

| Model | Scaled Residuals | | | | p-Value | AIC | BMC (ppm) | BMCL (ppm) |
| --- | --- | --- | --- | --- | --- | --- | --- | --- |
|  | At 0 ppm | At 75 ppm | At 150 ppm | At 300 ppm |  |  |  |  |
| Exponential 2 | -1.00555 | 1.05301 | 0.18291 | -0.25633 | 0.3233 | 343.148 | 178.90 | 143.67 |
| Exponential 3 | -1.00555 | 1.05301 | 0.18291 | -0.25633 | 0.3233 | 343.148 | 178.90 | 143.67 |
| Exponential 4 | -0.21520 | 0.67958 | -0.60015 | 0.13577 | 0.3447 | 343.783 | 116.57 | 59.20 |
| Exponential 5 | -0.21521 | 0.67959 | -0.60013 | 0.13575 | 0.3447 | 343.783 | 116.57 | 59.20 |
| **Hill** | **-0.17420** | **0.63204** | **-0.61893** | **0.16110** | **0.3581** | **343.734** | **113.72** | **51.91*** |
| Polynomial Degree 3 | -0.70041 | 0.98575 | -0.07781 | -0.20753 | 0.4654 | 342.419 | 155.20 | 117.30 |
| Polynomial Degree 2 | -0.70041 | 0.98575 | -0.07781 | -0.20753 | 0.4654 | 342.419 | 155.20 | 117.30 |
| Power | -0.70041 | 0.98575 | -0.07781 | -0.20753 | 0.4654 | 342.419 | 155.20 | 117.31 |
| Linear | 0.98575 | 0.98575 | -0.07781 | -0.20753 | 0.4654 | 342.419 | 155.20 | 117.30 |

*BMCL value used in Table 5

Table 2O. Summary table for MucilAir benchmark concentration modelling – Test Chemical: TES; Endpoint: TEER

| Model | Scaled Residuals | | | | p-Value | AIC | BMC (ppm) | BMCL (ppm) |
| --- | --- | --- | --- | --- | --- | --- | --- | --- |
|  | At 0 ppm | At 75 ppm | At 150 ppm | At 300 ppm |  |  |  |  |
| Exponential 2 | 0.14170 | -0.99504 | 1.57533 | 1.42806 | 0.0551 | 808.295 | 17.17 | 11.50 |
| Exponential 3 | 0.14170 | -0.99503 | 1.57533 | 1.42806 | 0.0551 | 808.295 | 17.17 | 11.50 |
| **Exponential 4** | **0.00595** | **-0.12381** | **0.62951** | **-0.51185** | **0.4105** | **805.174** | **10.63** | **4.72*** |
| Exponential 5 | 0.00594 | -0.12383 | 0.62952 | -0.51183 | 0.4105 | 805.174 | 10.63 | 4.72 |
| Hill | 0.00150 | -0.16379 | 0.41621 | -0.25413 | 0.6065 | 804.762 | 4.98 | 0.00 |
| Polynomial Degree 3 | 3.40824 | -3.54884 | -1.71676 | 1.74559 | <0.0001 | 844.191 | 132.75 | 102.66 |
| Polynomial Degree 2 | 3.40824 | -3.54884 | -1.71676 | 1.74559 | <0.0001 | 844.191 | 132.75 | 102.66 |
| Power | 3.40824 | -3.54884 | -1.71676 | 1.74559 | <0.0001 | 844.191 | 132.75 | 102.67 |
| Linear | -3.54884 | -3.54884 | -1.71676 | 1.74559 | <0.0001 | 844.191 | 132.75 | 102.66 |

*BMCL value used in Table 5

Table 2P. Summary table for MucilAir benchmark concentration modelling – Test chemical: TMS; Endpoint: LDH release

| Model | Scaled Residuals | | | | p-Value | AIC | BMC (ppm) | BMCL (ppm) |
| --- | --- | --- | --- | --- | --- | --- | --- | --- |
|  | At 0 ppm | At 25 ppm | At 100 ppm | At 300 ppm |  |  |  |  |
| Exponential 2 | -0.69028 | -0.33138 | 0.85921 | -0.08948 | 0.5097 | 328.045 | 216.46 | 190.59 |
| Exponential 3 | -0.69186 | -0.33301 | 0.85722 | -0.08854 | 0.5097 | 328.045 | 216.49 | 190.59 |
| **Exponential 4** | **0.05004** | **-0.07114** | **0.02252** | **-0.00155** | **0.9284** | **328.705** | **137.03** | **67.10*** |
| Exponential 5 | 0.03825 | -0.06852 | 0.03725 | -0.00834 | NA | 330.705 | 138.06 | 67.07 |
| Hill | -0.00169 | 0.00211 | -0.00292 | 0.00246 | NA | 330.697 | 135.62 | 64.16 |
| Polynomial Degree 3 | -0.04629 | -0.08336 | 0.17382 | -0.05148 | 0.9792 | 326.739 | 147.84 | 112.64 |
| Polynomial Degree 2 | -0.04628 | -0.08335 | 0.17382 | -0.05151 | 0.9792 | 326.739 | 147.84 | 112.64 |
| Power | -0.04628 | -0.08335 | 0.17382 | -0.05150 | 0.9792 | 326.739 | 147.84 | 112.64 |
| Linear | -0.08336 | -0.08336 | 0.17381 | -0.05149 | 0.9792 | 326.739 | 147.84 | 112.64 |

*BMCL value used in Table 5

Table 2Q. Summary table for MucilAir benchmark concentration modelling – Test chemical: TMS; Endpoint: PrestoBlue

| Model | Scaled Residuals | | | | p-Value | AIC | BMC (ppm) | BMCL (ppm) |
| --- | --- | --- | --- | --- | --- | --- | --- | --- |
|  | At 0 ppm | At 25 ppm | At 100 ppm | At 300 ppm |  |  |  |  |
| Exponential 2 | 2.03462 | -1.70413 | -0.94951 | 0.66560 | 0.0057 | 200.176 | 102.17 | 65.61 |
| Exponential 3 | 2.03464 | -1.70414 | -0.94952 | 0.66561 | 0.0057 | 200.176 | 102.17 | 65.61 |
| Exponential 4 | 0.07719 | -0.27251 | 1.26755 | -1.07223 | 0.0823 | 194.875 | 9.94 | 4.86 |
| Exponential 5 | 0.07534 | -0.26770 | 1.26688 | -1.07452 | 0.0823 | 194.875 | 9.91 | 4.86 |
| **Hill** | **0.06228** | **-0.41392** | **0.95927** | **-0.60764** | **0.2190** | **193.367** | **8.23** | **2.21*** |
| Polynomial Degree 3 | 2.22458 | -1.58486 | -1.15769 | 0.51797 | 0.0034 | 201.246 | 129.95 | 90.07 |
| Polynomial Degree 2 | 2.22458 | -1.58486 | -1.15769 | 0.51797 | 0.0034 | 201.246 | 129.95 | 90.07 |
| Power | 2.22458 | -1.58486 | -1.15769 | 0.51797 | 0.0034 | 201.246 | 129.95 | 90.07 |
| Linear | -1.58486 | -1.58486 | -1.15769 | 0.51797 | 0.0034 | 201.246 | 129.95 | 90.07 |

*BMCL value used in Table 5

Table 2R. Summary table for MucilAir benchmark concentration modelling – Test chemical: TMS; Endpoint: IL-6

| Model | Scaled Residuals | | | | p-Value | AIC | BMC (ppm) | BMCL (ppm) |
| --- | --- | --- | --- | --- | --- | --- | --- | --- |
|  | At 0 ppm | At 25 ppm | At 100 ppm | At 300 ppm |  |  |  |  |
| Exponential 2 | -2.88014 | 1.07642 | 2.04907 | -0.35494 | 0.00041 | 643.1291 | 199.26 | 170.01 |
| Exponential 3 | -2.88024 | 1.07632 | 2.04897 | -0.35498 | 0.00041 | 643.1291 | 199.26 | 170.01 |
| Exponential 4 | -0.53479 | 1.05579 | -0.93314 | 0.35186 | 0.11807 | 631.9893 | 32.89 | 14.75 |
| Exponential 5 | -0.53510 | 1.05589 | -0.93294 | 0.35169 | 0.11807 | 631.9893 | 32.90 | 14.75 |
| **Hill** | **-0.15867** | **0.52907** | **-0.79224** | **0.37069** | **0.29881** | **630.6261** | **23.49** | **10.42*** |
| Polynomial Degree 2 | -2.33747 | 1.41378 | 1.67021 | -0.63865 | 0.00288 | 639.2438 | 141.41 | 109.73 |
| Power | -2.33748 | 1.41376 | 1.67020 | -0.63865 | 0.00288 | 639.2438 | 141.41 | 109.73 |
| Linear | 1.41376 | 1.41376 | 1.67020 | -0.63860 | 0.00288 | 639.2438 | 141.41 | 109.73 |

*BMCL value used in Table 5

Table 2S. Summary table for MucilAir benchmark concentration modelling – Test chemical: TMS; Endpoint: CXCL-8 (IL-8

| Model | Scaled Residuals | | | | p-Value | AIC | BMC (ppm) | BMCL (ppm) |
| --- | --- | --- | --- | --- | --- | --- | --- | --- |
|  | At 0 ppm | At 25 ppm | At 100 ppm | At 300 ppm |  |  |  |  |
| Exponential 2 | -2.39543 | 1.30423 | 1.44334 | -0.34368 | 0.00531 | 1375.5497 | 250.39 | 197.53 |
| Exponential 3 | -2.39557 | 1.30410 | 1.44327 | -0.34347 | 0.00531 | 1375.5497 | 250.40 | 197.53 |
| Exponential 4 | -0.09446 | 0.29341 | -0.99236 | 0.72932 | 0.20129 | 1368.7047 | 25.89 | 10.78 |
| Exponential 5 | -0.09468 | 0.29381 | -0.99238 | 0.72912 | 0.20129 | 1368.7047 | 25.90 | 10.78 |
| **Hill** | **-0.04728** | **0.28205** | **-0.65786** | **0.38052** | **0.41554** | **1367.7345** | **24.94** | **6.15*** |
| Polynomial Degree 2 | -2.11879 | 1.46218 | 1.24832 | -0.51107 | 0.01067 | 1374.1520 | 213.28 | 152.22 |
| Power | -2.11879 | 1.46218 | 1.24832 | -0.51108 | 0.01067 | 1374.1520 | 213.28 | 152.22 |
| Linear | 1.46218 | 1.46218 | 1.24832 | -0.51108 | 0.01067 | 1374.1520 | 213.28 | 152.22 |

*BMCL value used in Table 5

Table 2T. Summary table for MucilAir benchmark concentration modelling – Test chemical: TMS; Endpoint: AAA

| Model | Scaled Residuals | | | | p-Value | AIC | BMC (ppm) | BMCL (ppm) |
| --- | --- | --- | --- | --- | --- | --- | --- | --- |
|  | At 0 ppm | At 25 ppm | At 100 ppm | At 300 ppm |  |  |  |  |
| Exponential 2 | 0.47154 | -0.33970 | -0.46344 | -0.33572 | 0.6704 | 577.627 | 84.40 | 54.45 |
| Exponential 3 | 0.47148 | -0.33970 | -0.46337 | -0.33573 | 0.6704 | 577.627 | 84.40 | 54.45 |
| Exponential 4 | 0.13233 | -0.22303 | 0.11696 | 0.73307 | 0.7751 | 578.909 | 63.48 | 33.16 |
| Exponential 5 | 0.13198 | -0.22278 | 0.11764 | 0.73288 | 0.7751 | 578.909 | 63.46 | 33.16 |
| **Hill** | **0.05744** | **-0.11456** | **0.08147** | **0.38122** | **0.8778** | **578.851** | **60.13** | **26.99*** |
| Polynomial Degree 3 | 1.17389 | -0.24211 | -1.42794 | -0.49859 | 0.1470 | 580.662 | 143.56 | 111.23 |
| Polynomial Degree 2 | 1.17389 | -0.24211 | -1.42794 | -0.49859 | 0.1470 | 580.662 | 143.56 | 111.23 |
| Power | 1.17389 | -0.24211 | -1.42794 | -0.49859 | 0.1470 | 580.662 | 143.56 | 111.23 |
| Linear | -0.24211 | -0.24211 | -1.42794 | 0.49616 | 0.1470 | 580.662 | 143.56 | 111.23 |

*BMCL value used in Table 5

Table 2U. Summary table for MucilAir benchmark concentration modelling – Test chemical: TMS; Endpoint: CBF

| Model | Scaled Residuals | | | | p-Value | AIC | BMC (ppm) | BMCL (ppm) |
| --- | --- | --- | --- | --- | --- | --- | --- | --- |
|  | At 0 ppm | At 25 ppm | At 100 ppm | At 300 ppm |  |  |  |  |
| Exponential 2 | 0.37545 | 0.18811 | -0.71710 | 0.16732 | 0.6968 | 342.294 | 211.31 | 164.96 |
| Exponential 3 | -0.08576 | 0.09996 | -0.01483 | 0.00069 | 0.8945 | 343.589 | 254.61 | 169.93 |
| Exponential 4 | 0.45163 | 0.17188 | -0.91400 | 0.29067 | 0.2807 | 344.735 | 199.20 | 152.23 |
| Exponential 5 | -0.08532 | 0.10693 | -0.02227 | 0.00167 | NA | 345.590 | 250.61 | 177.96 |
| Hill | -0.11184 | 0.11265 | 0.00014 | -0.00086 | NA | 345.596 | 118.67 | 105.00 |
| Polynomial Degree 3 | -0.04582 | 0.06272 | -0.01748 | 0.00061 | 0.9968 | 341.577 | 257.02 | 154.98 |
| **Polynomial Degree 2** | **-0.06316** | **0.12074** | **-0.06383** | **0.00625** | **0.9887** | **341.594** | **248.19** | **154.80*** |
| Power | -0.09121 | 0.10293 | -0.01240 | 0.00068 | 0.8902 | 343.590 | 251.22 | 154.84 |
| Linear | 0.17186 | 0.17186 | -0.91331 | 0.29010 | 0.5594 | 342.733 | 199.25 | 144.64 |

*BMCL value used in Table 5

Table 2V. Summary table for MucilAir benchmark concentration modelling – Test chemical: TMS; Endpoint: TEER

| Model | Scaled Residuals | | | | p-Value | AIC | BMC (ppm) | BMCL (ppm) |
| --- | --- | --- | --- | --- | --- | --- | --- | --- |
|  | At 0 ppm | At 25 ppm | At 100 ppm | At 300 ppm |  |  |  |  |
| Exponential 2 | 1.78981 | -2.04160 | -0.13537 | 0.50442 | 0.0171 | 904.224 | 197.69 | 101.75 |
| Exponential 3 | 1.78981 | -2.04161 | -0.13536 | 0.50444 | 0.0171 | 904.224 | 197.68 | 101.75 |
| **Exponential 4** | **0.04633** | **-0.22168** | **1.26291** | **-1.08757** | **0.0889** | **900.976** | **20.32** | **11.88*** |
| Exponential 5 | 0.00024 | -0.00018 | 1.18245 | -1.18274 | NA | 902.943 | 20.31 | 11.91 |
| Hill | 0.02239 | -0.35292 | 1.15743 | -0.82689 | 0.1393 | 900.268 | 19.67 | 0.00 |
| Polynomial Degree 3 | 2.12837 | -1.93079 | -0.53772 | 0.34014 | 0.0095 | 905.391 | 255.85 | 174.03 |
| Polynomial Degree 2 | 2.12837 | -1.93079 | -0.53772 | 0.34014 | 0.0095 | 905.391 | 255.85 | 174.03 |
| Power | 2.12837 | -1.93079 | -0.53772 | 0.34014 | 0.0095 | 905.391 | 255.85 | 174.03 |
| Linear | -1.93079 | -1.93079 | -0.53772 | 0.34014 | 0.0095 | 905.391 | 255.85 | 174.03 |

*BMCL value used in Table 5
